# Discovery of a cooperative mode of inhibiting RIPK1 kinase

**DOI:** 10.1038/s41421-021-00278-x

**Published:** 2021-06-01

**Authors:** Huyan Meng, Guowei Wu, Xinsuo Zhao, Anhui Wang, Dekang Li, Yilun Tong, Taijie Jin, Ye Cao, Bing Shan, Shichen Hu, Ying Li, Lifeng Pan, Xiaoxu Tian, Ping Wu, Chao Peng, Junying Yuan, Guohui Li, Li Tan, Zhaoyin Wang, Ying Li

**Affiliations:** 1 Interdisciplinary Research Center on Biology and Chemistry, Shanghai Institute of Organic Chemistry, Chinese Academy of Sciences, Shanghai, China; 2 University of Chinese Academy of Sciences, Beijing, China; 3 Dalian Institute of Chemical Physics, Chinese Academy of Sciences, Dalian, Liaoning China; 4 Shanghai Institute of Organic Chemistry, Chinese Academy of Sciences, Shanghai, China; 5 National Facility for Protein Science, Zhangjiang Lab, Shanghai Advanced Research Institute, Chinese Academy of Sciences, Shanghai, China; 6 Department of Cell Biology, Harvard Medical School, Boston, MA USA

**Keywords:** Cell death, Cell signalling

## Abstract

RIPK1, a death domain-containing kinase, has been recognized as an important therapeutic target for inhibiting apoptosis, necroptosis, and inflammation under pathological conditions. RIPK1 kinase inhibitors have been advanced into clinical studies for the treatment of various human diseases. One of the current bottlenecks in developing RIPK1 inhibitors is to discover new approaches to inhibit this kinase as only limited chemotypes have been developed. Here we describe Necrostatin-34 (Nec-34), a small molecule that inhibits RIPK1 kinase with a mechanism distinct from known RIPK1 inhibitors such as Nec-1s. Mechanistic studies suggest that Nec-34 stabilizes RIPK1 kinase in an inactive conformation by occupying a distinct binding pocket in the kinase domain. Furthermore, we show that Nec-34 series of compounds can synergize with Nec-1s to inhibit RIPK1 in vitro and in vivo. Thus, Nec-34 defines a new strategy to target RIPK1 kinase and provides a potential option of combinatorial therapy for RIPK1-mediated diseases.

## Introduction

Necroptosis is a form of regulated inflammatory necrotic cell death that can be activated in a wide range of human inflammatory or degenerative diseases such as amyotrophic lateral sclerosis, multiple sclerosis, Gaucher’s disease and Alzheimer’s disease^1–8^. In cells stimulated by tumor necrosis factor (TNF) α under certain conditions, the kinase activity of receptor-interacting protein kinase 1 (RIPK1) is activated^8–10^, which allows RIPK1 to bind to RIPK3 to promote its activation^11–13^. The activated RIPK3 in turn mediates the phosphorylation of MLKL (mixed-lineage kinase domain-like pseudokinase) to promote necroptosis^14^. The regulated nature of necroptosis was first discovered by studies using specific chemical inhibitors of RIPK1, such as necrostatin-1 (Nec-1) and its optimized analog, Nec-1s^9,10,15^. Nec-1/Nec-1s have been widely applied in the research to investigate the mechanism of necroptosis and animal models of human diseases, which revealed the role of RIPK1 kinase activity as a crucial mediator of cell death and inflammation in many human diseases^7,16^.

RIPK1 kinase possesses a unique allosteric pocket behind the ATP-binding site that is highly amenable to the development of small-molecule inhibitors^17^. All three published necrostatins, Nec-1s, Nec-3 and Nec-4, bind to this allosteric pocket on RIPK1 kinase domain to prevent the transition of a proximate DLG-motif in “DLG-out” inactivate conformation to “DLG-in” active conformation. Thus, Nec-1s, Nec-3 and Nec-4 belong to type-III kinase inhibitors, which generally exhibit superior specificity than other classes of kinase inhibitors^18^. Similar RIPK1 inhibitors have been developed, including GSK2982772 developed by GlaxoSmithKline and DNL747 by Denali Therapeutics, which have successfully completed Phase I clinical trials^19,20^ with acceptable safety profiles. Indeed, the development of potent and specific RIPK1 inhibitors offers hope that inhibition of RIPK1 may provide a successful strategy for the treatment of many chronic degenerative or auto-inflammatory diseases, where the selectivity and safety of a drug for chronic dosing are paramount.

In this study, we described a new mechanism of inhibiting RIPK1 by Necrostatin-34 (Nec-34). We show that Nec-34 and its active analogs are also direct inhibitors of RIPK1 kinase, but with a distinct inhibitory mechanism. We interrogated the mechanism of Nec-34 using photo-affinity probe strategy, Gaussian accelerated Molecular Dynamics (GaMD) enhanced sampling simulations, Limited proteolysis-mass spectrometry (LiP-MS) and hydrogen deuterium exchange-mass spectrometry (HDX-MS) analysis, which allowed us to define a unique Nec-34-binding pocket in RIPK1 kinase domain distinct from that of Nec-1s. Interestingly, we found that Nec-34 series of compound could synergize with Nec-1s in inhibiting RIPK1 kinase both in vitro and in vivo. The discovery of Nec-34 defines a new binding mode for inhibiting RIPK1 and provides a novel combinatorial treatment option for RIPK1-mediated pathologies.

## Results

### Identification of Nec-34 as an inhibitor of necroptosis

**Fig. 1:**
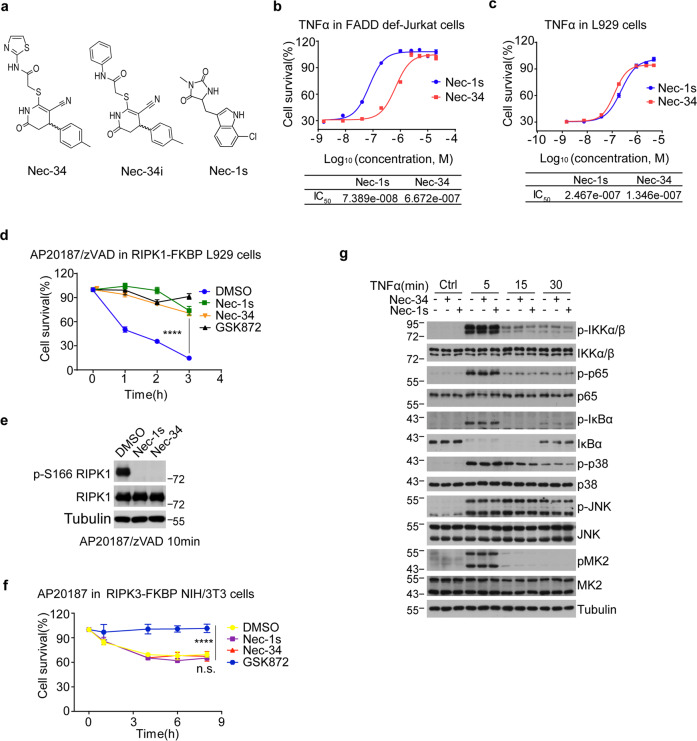
Nec-34 inhibits necroptosis of human and mouse cells. **a** Chemical structures of Nec-34, Nec-34i and Nec-1s. **b**, **c** FADD-deficient Jurkat cells (**b**) and L929 cells (**c**) were pretreated with different concentrations of Nec-1s or Nec-34 for 30 min and then TNFα was added for additional 16 h. **d**, **e** RIPK1 KO L929 cells were infected with retrovirus expressing Flag-tagged WT mRIPK1-FKBP using the Tet-On Advanced Inducible Expression System. RIPK1-reconstituted L929 cells were treated with 1 μg/mL doxycycline for 48 h to induce RIPK1 expression. The cells were then treated with 10 μM Nec-1s, Nec-34 or GSK872 for 30 min and then 1 nM AP20187 and 50 μM zVAD.fmk were added for indicated periods of time (**d**). The RIPK1 kinase activity was analyzed by western blotting with the indicated antibodies (**e**). **f** RIPK3-FKBP NIH/3T3 cells were pretreated with 10 μM Nec-1s, Nec-34 or GSK872 for 30 min and then 2 nM AP20187 was added for additional periods of time. **g** L929 cells were pretreated with 10 μM Nec-1s or Nec-34 for 30 min and then 100 ng/mL TNFα was added for indicated periods of time. The cell lysates were analyzed by phosphorylated and total IKKα/β, p65, IκBα, p38, JNK, MK2 and actin as indicated. The cell death in **b**–**d** and **f** were measured by CellTiter-Glo assays. The results shown depict the means ± SEM of *n* = 3 independent biological experiments. *P*-values were calculated by two-tailed Student’s *t*-test (n.s., not significant; ****P < 0.0001).

Nec-34 was identified as a potent inhibitor of necroptosis from two parallel cell-based phenotypical screens of TNFα-induced necroptosis in FADD-deficient Jurkat cells and zVAD.fmk-induced necroptosis in L929 cells against a chemical library with around 500,000 compounds (Fig. 1a). A structurally close inactive analog of Nec-34, Nec-34i, was identified in the screen, suggesting the specificity of Nec-34 in interacting with its target (Fig. 1a). Nec-34 is structurally distinct from the known potent necroptosis inhibitor Nec-1s. We assessed the activity of Nec-34 on commonly used cellular models of apoptosis and necroptosis. We found that Nec-34, but not Nec-34i, inhibited necroptosis induced by TNFα, or TNFα in combination with small-molecule IAP antagonist SM-164 or TAK1 inhibitor (5Z)-7-oxozeaenol (5Z7) in both FADD-deficient Jurkat cells (Supplementary Fig. S1a) and L929 cells (Supplementary Fig. S1b). On the other hand, Nec-34 did not inhibit canonical apoptosis induced by TNFα/CHX (Supplementary Fig. S1c, d). Dose-response studies suggested that the IC_50_ values of Nec-34 were in the same concentration range with that of Nec-1s in the common cell-based models using both murine and human cell lines (Fig. 1b, c and Supplementary Fig. S1e, f). Thus, Nec-34 is a novel inhibitor of necroptosis.

We further evaluated the inhibitory activity of Nec-34 on cell death induced by forced dimerization of RIPK1 or RIPK3. RIPK1 knockout L929 cells were transfected with an expressing vector encoding an inducible and dimerizable RIPK1 fused with FKBP at the C-terminus. Nec-34 effectively inhibited necroptosis induced by the addition of AP20187 and zVAD.fmk (Fig. 1d). Furthermore, Nec-34 inhibited the dimerization-induced RIPK1 activation as examined by phosphorylation of Ser166 (p-S166) of RIPK1, a biomarker for RIPK1 activation^1^ (Fig. 1e). In contrast, Nec-34 was unable to prevent the cell death or RIPK3 phosphorylation induced by forced dimerization of RIPK3, which can be inhibited by a RIPK3 inhibitor GSK872^21^ (Fig. 1f and Supplementary Fig. S1g). Moreover, Nec-34 had no effect on the early activation signatures of NF-κB and MAPK pathways, including phosphorylation of IKKα/β, p65, p38, JNK, MK2, IκBα, and degradation of IκBα, upon treatment with TNFα in mouse L929 cells (Fig. 1g) or human FADD-deficient Jurkat cells (Supplementary Fig. S1h). Taken together, these results suggest that Nec-34 is an inhibitor of RIPK1 kinase.

### Nec-34 disrupts the formation of complex II

**Fig. 2:**
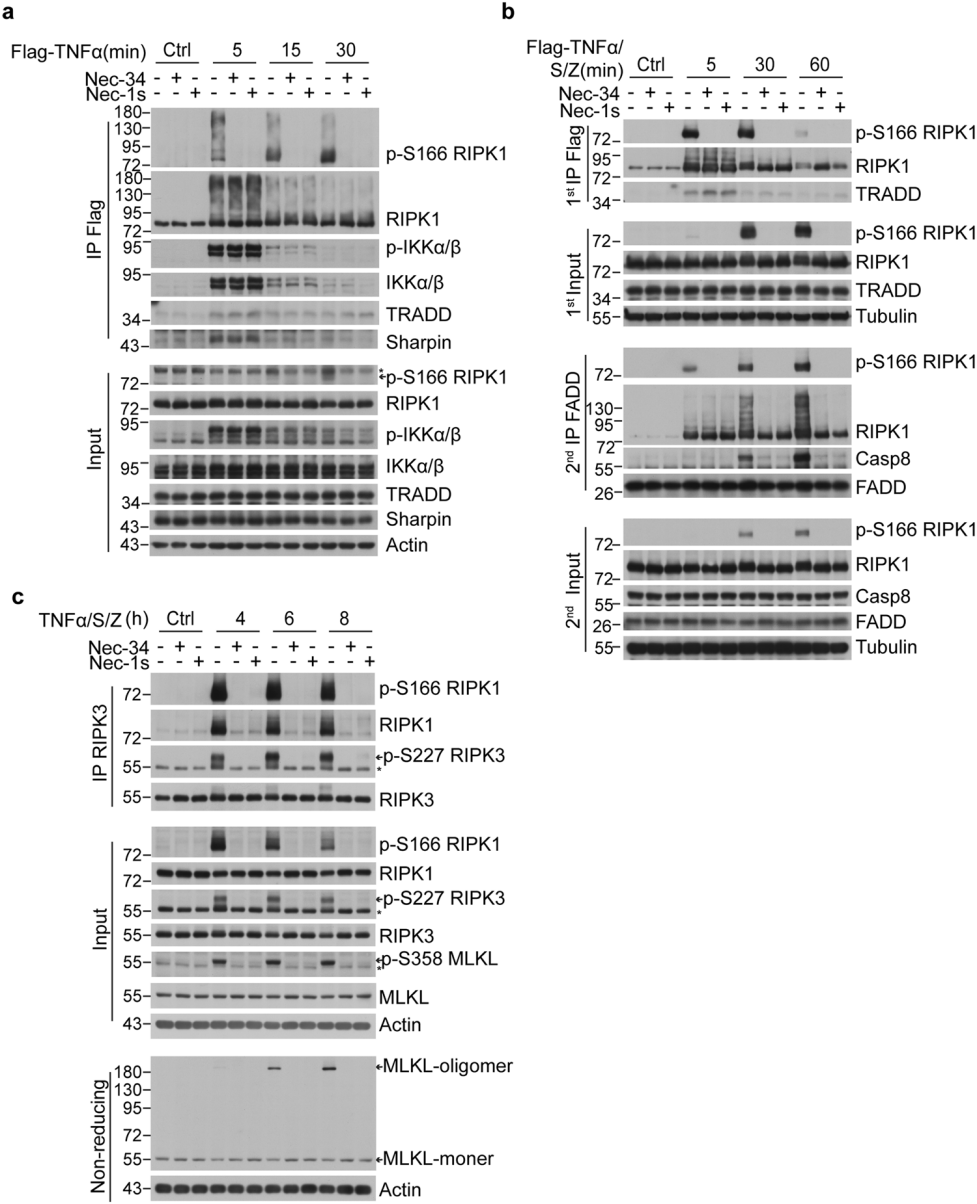
Nec-34 inhibits the activity of RIPK1 and disrupts the formation of complex II. **a** L929 cells were pretreated with 10 μM Nec-1s or Nec-34 for 30 min and then 100 ng/mL Flag-TNFα was added for indicated time points. The cells were lysed with 0.5% Nonidet P-40 buffer and cell lysates were immunoprecipitated with anti-Flag antibody-conjugated agarose. All immunoprecipitated complexes and whole-cell lysates were analyzed by western blotting with the indicated antibodies. **b** MEFs were pretreated with 10 μM Nec-1s or Nec-34 for 30 min and then pretreated with 100 nM SM-164 (S) for 2 h and 50 μM zVAD.fmk (Z) for 30 min, and 100 ng/mL Flag-TNFα was then added for 5, 30, and 60 min. The cells were lysed with 0.5% Nonidet P-40 buffer. The cell lysates were collected and sequentially immunoprecipitated with anti-Flag antibody-conjugated agarose and anti-FADD antibody. First immunoprecipitation (IP): TNFR1 complex I was immunoprecipitated using anti-Flag antibody-conjugated agarose. Second IP: the supernatants after the first IP were then immunoprecipitated with anti-FADD antibody. The immunoprecipitated complexes and whole-cell lysates were analyzed by western blotting with the indicated antibodies. **c** FADD-deficient Jurkat cells were pretreated with 10 μM Nec-1s or Nec-34 for 30 min and then treated with 50 ng/mL TNFα for indicated time points. The cells were lysed with 0.5% Nonidet P-40 buffer and cell lysates were immunoprecipitated with anti-RIPK3 antibody. All immunoprecipitated complexes and whole-cell lysates were analyzed by western blotting with the indicated antibodies.

Upon stimulation with TNFα, RIPK1 is recruited rapidly to TNFR1 to form the TNF-signaling complex (TNF-RSC, or complex I) along with other components such as TRADD, Sharpin — a key component of the M1-ubiquitinating enzyme LUBAC complex, and IKKα/β — inhibitor of nuclear factor κ-B kinase subunit α/β^22^. We characterized the effect of Nec-34 on the formation of complex I in TNFα-stimulated L929 cells and found that Nec-34 had no effect on the recruitment and ubiquitination of RIPK1 or the recruitment of TRADD, Sharpin or IKKα/β in complex I, whereas RIPK1 kinase activation, indicated by p-S166 RIPK1, was strongly blocked by Nec-34, as by Nec-1s (Fig. 2a). A similar result was obtained with necroptosis of TNFα-stimulated FADD-deficient Jurkat cells (Supplementary Fig. S2a).

Since RIPK1 activation is indispensable for the formation of complex II, we next analyzed the effect of Nec-34 on the interaction between RIPK1 and FADD^23^, a critical step for the formation of complex II. We examined the formation of complex I and complex II in MEFs stimulated with Flag-TNFα/SM-164/zVAD.fmk by sequential immunoprecipitation with anti-Flag and anti-FADD antibodies. The activation of RIPK1 as indicated by the p-S166 of RIPK1, was abolished by Nec-34 in both complexes. Furthermore, the binding of RIPK1/FADD or caspase-8/FADD was blocked by Nec-34, as by Nec-1s, while the recruitment of RIPK1 into TNF-complex I was unaffected (Fig. 2b).

We also analyzed the effect of Nec-34 on the interaction of activated RIPK1 with RIPK3 in FADD-deficient Jurkat cells stimulated by TNFα. We found that the interaction of RIPK1 with RIPK3, the downstream activation of RIPK3 and MLKL, and the oligomerization of MLKL were all blocked by Nec-34, as well as Nec-1s (Fig. 2c). A consistent result was also observed in HT29 cells stimulated by TNFα/SM-164/zVAD.fmk (Supplementary Fig. S2b). Since Nec-34 had no effect on the cell death induced by forced dimerization of RIPK3 (Fig. 1f), these results suggest that Nec-34 may block TNFα-induced complex II formation by inhibiting the activation of RIPK1 kinase.

### Nec-34 is a direct inhibitor of RIPK1 kinase

**Fig. 3:**
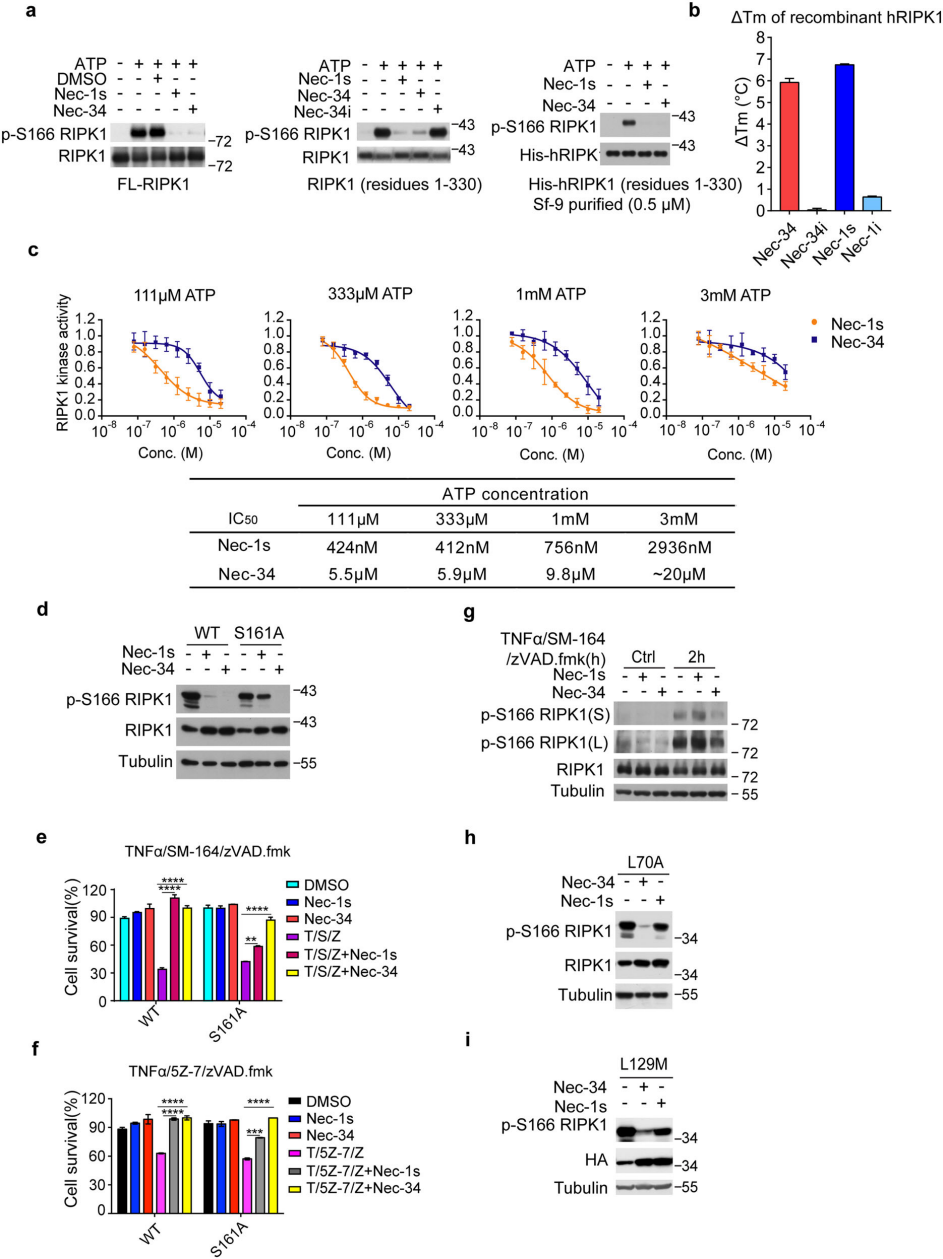
Nec-34 directly inhibits the kinase activity of RIPK1. **a** The in vitro kinase activity assay. 293T cells were transfected with HA-tagged FL-RIPK1 (left) or kinase domain of RIPK1 (residues 1–330) (middle) for 24 h. Cells were then lysed with Nonidet P-40 buffer 24 h after transfection. The cell lysates were immunoprecipitated with anti-HA antibody-conjugated agarose and divided equally into five or four parts. Divided immunocomplexes were pretreated with 20 μM Nec-1s, Nec-34, or Nec-34i for 10 min. Kinase reactions were initiated by 20 μM ATP, and the reactions were carried out at 30 °C for 30 min. Recombinant hRIPK1 (residues 1–330, 0.5 μM) purified from Sf-9 insect cells was pretreated with 10 μM Nec-1s or Nec-34 for 30 min as indicated. The kinase reactions were initiated by 200 μM ATP, and the reactions were carried out at 30 °C for 30 min (right). The samples were analyzed by western blotting with indicated antibodies. **b** The Tm value of recombinant hRIPK1 in Nec-34, Nec-34i, Nec-1s or Nec-1i treatment groups were compared to control group and presented as ΔTm for protein thermal shift assay. Recombinant hRIPK1 (residues 1–330, 2 μM) purified from Sf-9 cells was treated with indicated compounds for 1 h. Protein thermal stability was analyzed through the differential scanning calorimetry by real-time PCR and the Tm values were calculated by Protein Thermal Shift Software. Three replicates for each reaction were performed. **c** ADP-Glo™ Kinase Assay of hRIPK1. Recombinant hRIPK1 (residues 1–330, 1 μM) purified from Sf-9 cells was pretreated with different concentrations of Nec-1s, Nec-34 for 30 min as indicated. The kinase reactions were initiated by 111 μM, 333 μM, 1 mM and 3 mM ATP as indicated, and the reactions were carried out at 30 °C for 2 h, and then the amount of ADP produced during the kinase reaction was detected by ADP-Glo™ Reagent. **d** 293T cells were transfected with Flag-tagged WT-RIPK1 (residues 1–330), or S161A mutant for 24 h. Nec-34 or Nec-1s (10 μM) was added at 6 h after transfection. The cells were lysed with Nonidet P-40 buffer and analyzed by western blotting with indicated antibodies. **e**, **f** RIPK1-deficient Jurkat cells were infected with retrovirus expressing HA-tagged WT-hRIPK1 and HA-tagged hRIPK1 S161A, respectively, by the Tet-On Advanced Inducible Expression System. Reconstituted Jurkat cells were treated with 1 μg/mL doxycycline for 48 h to induce the expression of RIPK1. The cells were then pretreated with 100 nM SM-164 for 2 h (**e**) or 100 nM 5Z7 for 1 h (**f**), and then 100 ng/mL TNFα and 50 μM zVAD.fmk were added for additional 24 h. **g** Reconstituted hRIPK1 S161A Jurkat cells were pretreated with TNFα/SM-164/zVAD.fmk as indicated, and then were lysed with 0.5% Nonidet P-40 buffer and analyzed by western blotting with indicated antibodies. **h**, **i** 293T cells were transfected with HA-tagged RIPK1 (residues 1–330) L70A, or L129M mutant expression plasmids for 24 h. Nec-34 and Nec-1s (10 μM) were added 6 h after transfection. The cells were lysed with Nonidet P-40 buffer and analyzed by western blotting with indicated antibodies. The cell death in **e**, **f** were measured by CellTiter-Glo assays. The results shown depict the means ± SEM of *n* = 3 independent biological experiments. *P*-values were calculated by two-tailed Student’s *t*-test (**P < 0.01, ***P < 0.001, ****P < 0.0001).

RIPK1 comprises an N-terminal kinase domain, an intermediate domain, and a C-terminal death domain, and the kinase domain is the direct target of Nec-1s^10^ (Supplementary Fig. S3a). To explore whether Nec-34 inhibits RIPK1 kinase activity directly, we first purified full-length and kinase domain (residues 1–330) of RIPK1 from 293T overexpression system, and tested if Nec-34 could inhibit RIPK1 activation in biochemical kinase assays. We found that Nec-34 effectively blocked the activation of RIPK1 kinase in the context of both full-length and kinase domain only with similar potency compared to that of Nec-1s (Fig. 3a). Consistent result was also obtained with Sf-9 purified recombinant kinase domain of RIPK1 (Fig. 3a and Supplementary Fig. S3b). We further confirmed the binding of Nec-34 with purified recombinant RIPK1 kinase domain using fluorescence-based thermal shift assay^24^. As a positive control, Nec-1s significantly increased the thermal stability of recombinant hRIPK1. Similarly, the addition of Nec-34 also increased the melting temperature (Tm) of recombinant hRIPK1 by > 5 °C in the thermal stability assay (Fig. 3b and Supplementary Fig. S3c). Consistently, their inactive analogs Nec-1i or Nec-34i showed no effect in the same assay (Fig. 3b and Supplementary Fig. S3c). We directly verified the target engagement in a cellular format using the cellular thermal shift assay (CETSA), a robust tool to evaluate the binding of ligand to its target protein in cells and tissue samples in native status^25^. Nec-34 significantly stabilized RIPK1 in the soluble protein fraction at elevated temperatures, indicating that Nec-34 is able to bind endogenous RIPK1 in situ to increase its thermo-resistance (Supplementary Fig. S3d). These data suggest that Nec-34 directly inhibits RIPK1 activation by targeting the kinase activity of RIPK1.

Nec-1s binds to a hydrophobic pocket behind the ATP-binding site in RIPK1 kinase, which stabilizes RIPK1 in an inactive conformation^17^. To investigate the inhibitory mechanism of Nec-34, we next utilized an ADP-Glo Kinase Assay by measuring ADP production during autophosphorylation of RIPK1 in vitro. Interestingly, we found that both Nec-34 and Nec-1s were non-ATP-competitive at low ATP concentrations (< 1 mM) as indicated by the unaffected IC_50_ values, but became partially ATP-competitive at high concentrations of ATP (≥ 1 mM) (Fig. 3c). These results suggest that ATP competitiveness of Nec-34 in inhibiting RIPK1 kinase is similar to that of Nec-1s.

The members of RIP kinase family contain a conserved homologous kinase domain and additional different functional domains. We further analyzed the selectivity of Nec-34 across the RIP kinase family members by enzymatic kinase activity assay. Nec-34 showed no significant inhibition against commercially available RIPK2, RIPK3, RIPK4 and RIPK5 at 10 μM (Supplementary Fig. S3e), while the RIPK1 kinase activity was effectively blocked by Nec-34 at the same concentration in both biochemical and cellular assays (Figs. 1e and 3a, c). Given the significant role of RIPK3 in necroptosis, we further compared the effect of Nec-34 on RIPK1 and RIPK3 kinase activation in the same system. Nec-34 only inhibited RIPK1 but not RIPK3 autophosphorylation in 293T overexpression system (Supplementary Fig. S3f). Furthermore, Nec-34 prevented RIPK1 forced dimerization-induced kinase activation and cell death but not those of RIPK3 (Fig. 1d, f and Supplementary Fig. S1g). Taken together, these results indicate that Nec-34 exhibits remarkable selectivity toward RIPK1 kinase.

### Nec-34 inhibits RIPK1 kinase through a mechanism distinct from that of Nec-1s

Nec-1s specifically targets RIPK1 as a type-III kinase inhibitor by occupying the allosteric hydrophobic pocket adjacent to activation loop and stabilizing the kinase in a “DLG-out” inactive conformation^17^. Ser161 in the activation loop of RIPK1 is crucial for its binding with Nec-1s as the indole ring of Nec-1s forms a hydrogen bond (H-bond) with the hydroxyl oxygen of Ser161. Consistent with our previous finding^10^, the potency of Nec-1s in inhibiting RIPK1 autophosphorylation was impaired by S161A mutation as demonstrated by western blotting using p-S166 RIPK1 antibody (Fig. 3d). In contrast, Nec-34 effectively inhibited activation of both WT-RIPK1 and S161A mutant (Fig. 3d). Consistently, Nec-34 effectively inhibited necroptosis and RIPK1 activation in RIPK1-deficient Jurkat cells complemented with either WT or S161A RIPK1, while the inhibitory efficiency of Nec-1s was severely impaired by S161A mutation (Fig. 3e–g). Additionally, Nec-34 stabilized reconstituted S161A mutant RIPK1 as well as WT-RIPK1 in the soluble fraction under elevated temperatures as measured by CETSA, while the effect of Nec-1s was significantly impaired on S161A mutant versus WT (Supplementary Fig. S3g). Thus, S161 is important for Nec-1s, but not Nec-34, to inhibit RIPK1 kinase activity.

In addition, the indole ring of Nec-1s interacts with Leu70 and Leu129 of RIPK1 kinase through van der Waals contacts^17^. We found that L70A and L129M mutations dramatically weakened the inhibition of RIPK1 by Nec-1s, but not that by Nec-34 (Fig. 3h, i). Thus, Leu70 and Leu129 are important for Nec-1s, but not Nec-34, to inhibit RIPK1 kinase activity. Taken together, our results suggest that Nec-34 inhibits RIPK1 kinase through a mechanism distinct from that of Nec-1s.

### Characterization of Nec-34 binding mode on RIPK1 kinase

**Fig. 4:**
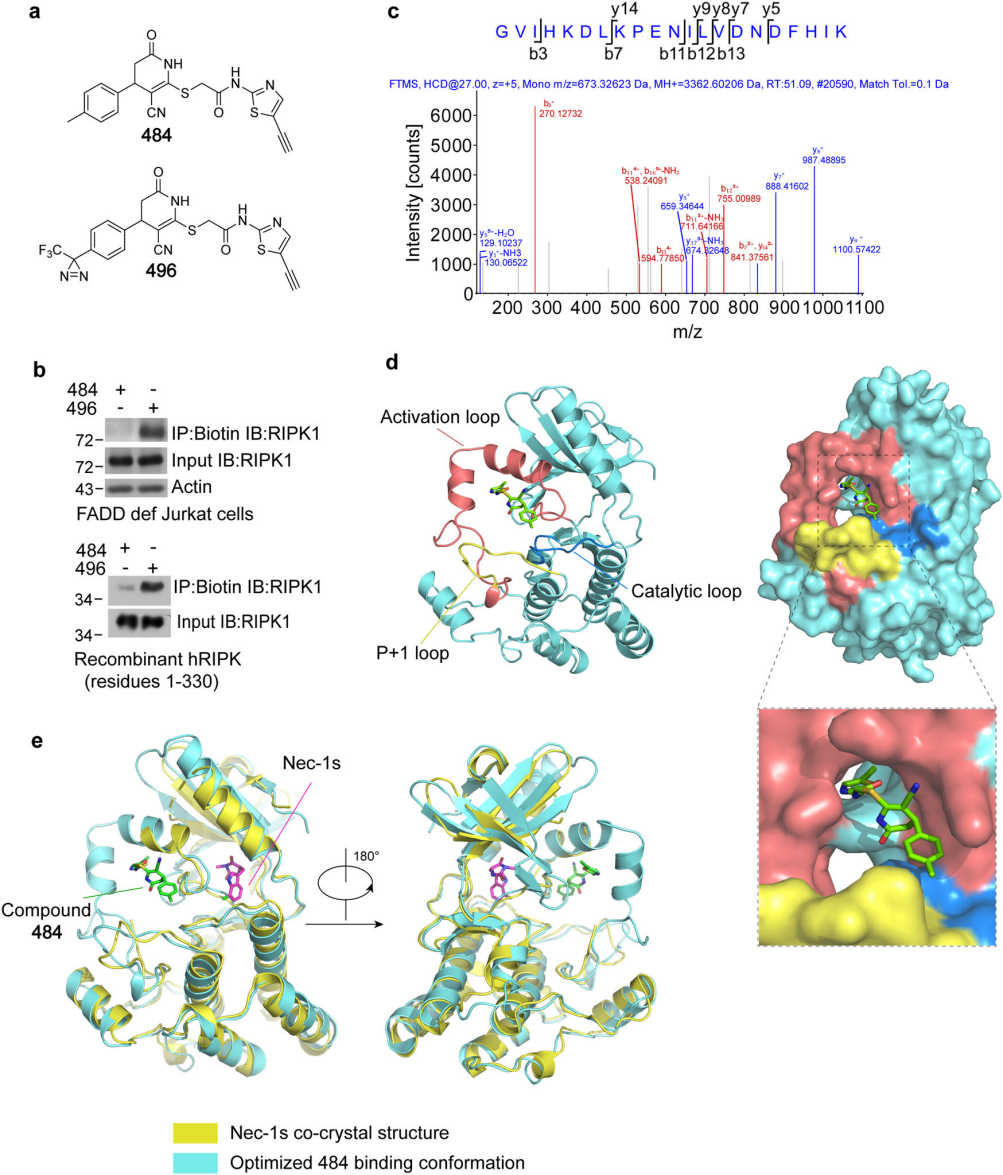
Identification of Nec-34-binding motifs in RIPK1 kinase. **a** The chemical structures of the photo-affinity probe compound 496 for Nec-34 and the photostable control compound 484. **b** FADD-deficient Jurkat cells were pretreated with 20 μM 484 or 496 for 1 h and then cells were lysed with Nonidet P-40 buffer. The cell lysates (upper blot) or purified hRIPK1 (residues 1–330, lower blot) were individually treated with 200 μM 484 or 496 for 30 min. Samples were subjected to photo-crosslinking assay and enriched by streptavidin-coupled beads. All isolated complexes and whole-cell lysates were analyzed by western blotting with indicated antibodies. **c** Identification of Nec-34-binding sites by mass spectrometry. hRIPK1 (residues 1–330, 50 μg) was labeled by incubating with photo-affinity probe 496 after UV-crosslinking. After click-chemistry reaction and biotin–streptavidin affinity purification, the enriched peptides of RIPK1 that bound with 496 were identified by mass spectrometry. MS/MS of a hRIPK1 peptide 133-GVIHKDLKPENILVDNDFHIK-153 crosslinked with biotinylated compound 496 is shown. The ion b3, b7, b11, b12, b13 are annotated with a mass shift +918.2587 Da. **d** Cartoon (left) and surface mode (right) of computationally optimized binding conformation of compound 484. The RIPK1 structure was obtained from RCSB PDB dataset (ID 6C4D) and the missing activation loop atoms were built with Modeler. The catalytic loop (Gly133–Asn143) was colored blue, the activation loop (Asp156–Glu196) was colored red, the P + 1 loop (His197–Thr206) was colored yellow, other parts of RIPK1 kinase domain were colored cyan. Compound 484 was shown in green sticks. **e** Alignment of computational binding mode of compound 484 in **d** with the co-crystal structure of RIPK1 and Nec-1s (PDB 4ITH). 484-bound RIPK1 was colored cyan, Nec-1s-bound RIPK1 was colored yellow. Compound 484 was shown in green sticks and Nec-1s was shown in magenta sticks.

We next used chemical biological techniques to further characterize the binding sites of Nec-34. Photo-affinity labeling followed by click chemistry enrichment allows visualization of bioactive small molecule binding with their targets^26^. To characterize the binding sites of Nec-34 in RIPK1, we designed and synthesized a photo-affinity probe of Nec-34, designated as compound 496, with diazirinyl and alkynyl modifications (Fig. 4a). Compound 484 was used as a photostable control for compound 496 (Fig. 4a). Upon irradiation with UV light, the diazirinyl of compound 496 would be converted into a carbene, which could insert into the X–H-bonds (X = C, N, S, O) of adjacent amino acid residues, resulting in a covalent adduct of 496 and RIPK1. The crosslinked RIPK1 can be further conjugated with biotin-azide via click reaction and enriched by affinity purification.

The synthesized compounds 496 and 484 were firstly tested for thermal shift assay and cell death assay. We found that both compounds 484 and 496 bound to RIPK1 kinase domain very well as indicated by the thermal shift assay (Supplementary Fig. S4a), and exhibited strong inhibition of necroptosis in FADD-deficient Jurkat cells (Supplementary Fig. S4b). Notably, only the photoreactive probe 496, but not the photostable control 484, was able to efficiently pulldown endogenous RIPK1 from FADD-deficient Jurkat cells and recombinant hRIPK1 purified from Sf-9 cells (Fig. 4b). We next performed mass spectrometry analysis on recombinant hRIPK1 pulled down using biotinylated compound 496 after photo-crosslinking. By searching MS/MS spectra against a human protein database under a control of FDR < 0.01 at peptide level, we identified four peptide spectrum-matches (PSMs) of a hRIPK1 peptide 133-GVIHKDLKPENILVDNDFHIK-153 with a mass shift +918.2587 Da (Fig. 4c and Supplementary Fig. S4c). The mass shift of this peptide is equal to the mass gain of biotinylated compound 496 (C39H45F3N10O7S3) after photo-crosslinking. As a control, compound 496-modified hRIPK1 peptides were not detected in the parallel preparation of the photo-crosslinked compound 484/hRIPK1 samples. However, the compound 496-specific-binding site could not be unambiguously assigned in this peptide by MS/MS spectra. The annotated MS/MS suggested that 133-GVIHKDL-139 of RIPK1 was the target region of compound 496 (Fig. 4c and Supplementary Fig. S4c).

We next used GaMD enhanced sampling simulations, a computational platform developed to assess the thermodynamics and kinetics of small molecule–target binding^27–30^, to characterize the binding mechanism of compound 484 in RIPK1 kinase. The simulation results showed that the residues located in the activation loop, P + 1 loop, and the catalytic loop, from His130 to Lys140, Ala160 to Asn170, and Asp180 to Asp200, had higher probabilities to form interactions with 484 (Supplementary Fig. S4d). Structural clustering of the 484 conformations in these regions was then performed to query plausible binding modes, yielding an optimized-binding conformation of 484 and an unprecedented binding pocket of RIPK1. As shown in Fig. 4d, the activation loop, P + 1 loop, and the catalytic loop of RIPK1 form a hydrophobic pocket into which 484 could nicely adapt. Alignment of this binding mode with the co-crystal structure of Nec-1s–RIPK1 (PDB 4ITH)^17^ revealed that 484 occupies a pocket adjacent to but distinct from the binding pocket of Nec-1s (Fig. 4e). Nec-1s inactivates RIPK1 by blocking its “DLG” motif from flipping-in, which consequently impedes the conformational transformation of the activation loop, and forces RIPK1 in a “DLG-out” inactive state. In contrast, 484 directly interacts with the activation loop, which would also hinder its movement, rendering RIPK1 to adapt a similar “DLG-out” inactive conformation but via a mechanism distinct from that of Nec-1s (Fig. 4d). In this binding mode, the thiazolyl ring of 484 is inserted into a hydrophobic cavity formed by activation loop (Fig. 4d). Notably, the conformation of 484 in this pocket closely resembled its single crystal structure (Supplementary Fig. S4e), with the σ_S_* orbital of the thiazolyl group (electron acceptor) interacting with the non-bonding orbital of the oxygen atom (electron donor) in the adjacent amide group. This kind of intramolecular noncovalent sulfur interaction would bias the stereo-orientation of the thiazolyl ring and favor the conformation of 484 in the S–O locked state (Supplementary Fig. S4e). Given that Nec-34i possesses a phenyl instead of thiazolyl (Fig. 1a), such S–O hypervalent interaction could facilitate 484 and Nec-34 to adapt favorable conformations binding to RIPK1. On the other hand, the 4-methylbenzyl group of 484 is surrounded by a hydrophobic groove formed by catalytic loop and P + 1 loop, with the 4-methyl protruding toward Ile135 and Lys137 (Supplementary Fig. S4f). As 496 possesses a photoreactive diazirinyl group at the same 4-position, and its labeling occurred in the same peptide (133-GVIHKDL-139), this predicted binding mode of 484 is reasonable. Finally, the activation loops in most kinases are known to be disordered in their inactive states^31^ and no structural information for the activation loop in RIPK1 kinase is available. Thus, the predicted binding of Nec-34 to the activation loop of RIPK1 kinase may also explain why it is difficult to obtain X-ray co-crystal structural information of Nec-34 in complex with RIPK1 kinase.

### Validation of Nec-34-binding pocket using LiP- and HDX-mass spectrometry

**Fig. 5:**
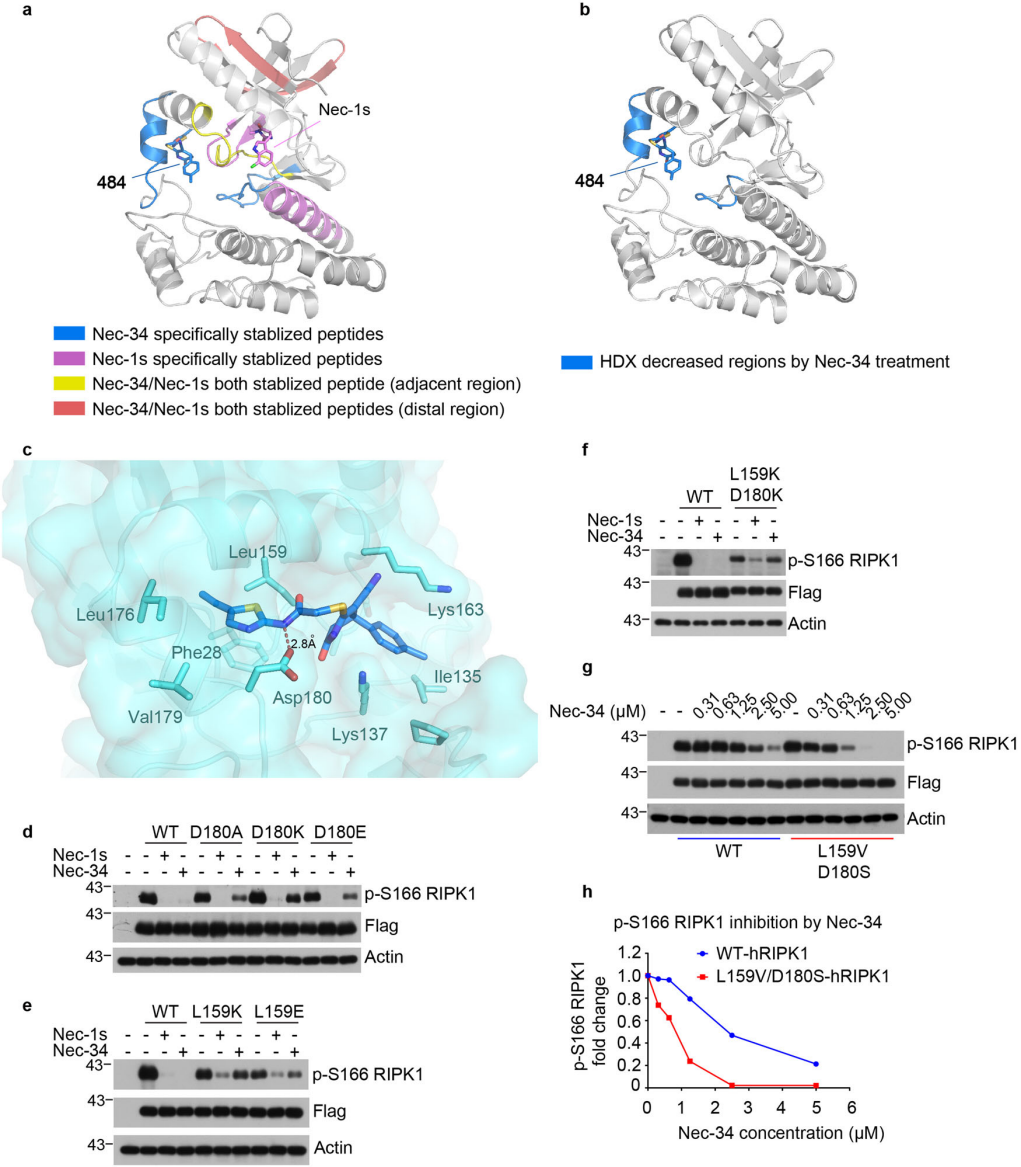
Validation of Nec-34-binding sites in RIPK1 kinase. **a** LiP-MS analysis of Nec-34-binding sites. Nec-34- and Nec-1s-stabilized peptides were mapped to aligned structure of 484 and Nec-1s with RIPK1 in Fig. 4e. 484-bound RIPK1 was colored gray. Peptides specifically stabilized by Nec-34 were colored blue. Peptides specifically stabilized by Nec-1s were colored magenta. The peptide stabilized by both Nec-34 and Nec-1s in the adjacent region was colored yellow. Peptides stabilized by both Nec-34 and Nec-1s in the distal region were colored red. **b** HDX-MS analysis of Nec-34-binding sites. Regions with decreased hydrogen deuterium exchange were mapped to the predicted binding model of 484 in Fig. 4d. HDX-decreased regions were colored blue. **c** A closed-up view of the 484-binding sites of RIPK1 according to the predicted binding mode of 484 in Fig. 4d. 484 was shown in blue sticks, the binding sites of 484 was shown in cyan sticks. The H-bond was shown in red dashed line. **d**–**f** 293T cells were transfected with Flag-tagged WT-RIPK1 (residues 1–330), Asp180 mutants of RIPK1 (**d**), Leu159 mutants of RIPK1 (**e**), or L159K/D180K double-mutant of RIPK1 (**f**) for 20 h. Cells were treated with 10 μM Nec-1s or Nec-34 12 h after transfection. Cells were lysed with Nonidet P-40 buffer and the cell lysates were analyzed by western blotting with indicated antibodies. **g** 293T cells were transfected with Flag-tagged WT-hRIPK1 (residues 1–330) or L159V/D180S double-mutant of hRIPK1 for 20 h, and treated with different concentrations of Nec-34 12 h after transfection. Cells were lysed with Nonidet P-40 buffer, and then cell lysates were analyzed by western blotting with indicated antibodies. **h** The signal intensities of bands from **g** were analyzed by ImageJ and showed by fold-change of p-S166 RIPK1 in different concentrations of Nec-34.

We further validated the binding mode of Nec-34 using LiP-MS and HDX-MS. LiP-MS is a label-free approach that can be used to identify the potential ligand-binding regions of target proteins^32–34^. This technique is based on the protection of proteinase K-mediated proteolysis in the binding region of the target protein by the compound, which can be characterized by mass spectrometry to define unique LiP-peptides that interact with the compound. Compared with LiP patterns in the non-bound control group, treatment with Nec-34 specifically increased the abundance of peptides 133-GVIHKDLKPENIL-145 and 173-HNELREVDGTA-183 (Fig. 5a and Supplementary Fig. S5a, blue-colored peptides), which were in the computationally predicted binding pocket of 484 (Fig. 4d). Furthermore, peptide 133-GVIHKDLKPENIL-145 was crosslinked by 496 (Fig. 4c). In comparison, treatment with Nec-1s led to increased abundance of peptides 118-IILEIIEGMAYLHG-131, a peptide known to interact with Nec-1s in the X-ray co-crystal structure, and 22-ELDSGGFGK-30, which is in a region not characterized in the X-ray co-crystal study^17^ (Fig. 5a and Supplementary Fig. S5a, b, magenta-colored peptides.). Interestingly, binding of Nec-1s and Nec-34 both increased the abundance of 154-IADLGLASFKMW-165 peptide, which is located between the binding pocket of Nec-1s in the X-ray co-crystal structure^17^ and the predicted 484-binding pocket (Fig. 5a and Supplementary Fig. S5a, b, yellow-colored peptide). Collectively, these results suggest that Nec-34 is able to bind to a distinct pocket on RIPK1 kinase adjacent to that of Nec-1s. In addition, two peptides 38-TQGLMIMK-45 and 78-LLGVIIEEGK-87, which located in the distal region of the binding pocket of Nec-34 and Nec-1s, were both stabilized by Nec-34 and Nec-1s (Fig. 5a and Supplementary Fig. S5a, b, red-colored peptides), suggesting that although Nec-34 and its analogs 484 and 496 occupy a binding pocket distinct from that of Nec-1s, they may be able to induce similar inhibitory conformational changes in the RIPK1 kinase.

We also used HDX-MS^35–37^, which measures isotopic exchange between amide hydrogens of the protein backbone and its surrounding solvent, to characterize the Nec-34 binding with RIPK1 kinase domain. The hydrogen deuterium exchange rate of backbone amide hydrogen is dependent on the dynamics of folded protein state and can be perturbed upon compound binding. HDX measurements of free RIPK1 in the presence of deuterium oxide from 30 s to 3000 s showed a dynamic exchange of deuterium in a region-specific manner (Supplementary Fig. S5c, d). As expected, the activation loop of RIPK1 showed rapid deuterium exchange within 30 s (Supplementary Fig. S5c, d), which is consistent with the overall flexible nature of this loop. Comparison of the global deuterium exchange spectra of free RIPK1 and Nec-34-bound RIPK1 revealed that treatment with Nec-34 rapidly decreased the deuterium exchange rate in two regions, 137-KDLKPEN-143 in the catalytic loop and 174-NELREVDG-181 in the activation loop (Fig. 5b and Supplementary Fig. S5e), which are almost identical to the two peptides specifically stabilized by Nec-34 as found by LiP-MS (Fig. 5a). Collectively, these results further support the predicted binding mode of 484 and demonstrate that Nec-34 and its analogs bind to a pocket distinct from that of Nec-1s.

### Verification of Nec-34-binding sites by site-directed mutagenesis studies

To further verify the Nec-34-binding mode proposed above with native RIPK1 in live cells, we performed site-directed mutagenesis studies. Based on this proposed binding mode, the amino acid residues Leu159, Asp180, and Lys137, may interact with 484 (Fig. 5c). Notably, the backbone amide of 484 forms a H-bond with the backbone carboxyl of Asp180 in the activation loop. Interestingly, although D180A, D180K or D180E mutations did not change the kinase activity of RIPK1 as indicated by the levels of autophosphorylation of RIPK1, these mutations all significantly decreased inhibition by Nec-34 (Fig. 5d). In contrast, the inhibition by Nec-1s was not affected by any of these mutations.

In addition, we also examined the possibility that certain hydrophobic interactions may be important for RIPK1’s binding with Nec-34. In our model, the thiazolyl ring of 484 adapts into a specific cavity surrounded by hydrophobic amino acids Phe28, Leu159, Leu176 and Val179 in the RIPK1 kinase domain (Figs. 4d and 5c). Interestingly, mutating Leu159 to a positively charged amino acid residue Lys slightly reduced RIPK1 kinase activity but resulted in a very significant decrease in the inhibition by Nec-34; while mutating Leu159 to a negatively charged amino acid residue Glu reduced RIPK1 kinase activity and also reduced inhibition by Nec-34 (Fig. 5e). L159K and L159E mutations also slightly weakened the inhibition by Nec-1s, but to a less extent (Fig. 5e). Moreover, L159K/D180K double mutation in RIPK1 reduced autophosphorylation of RIPK1 kinase and almost abolished the inhibition by Nec-34, while the inhibition by Nec-1s was only partially reduced (Fig. 5f). Taken together, these data provide further support for the distinct binding mode of Nec-34 compared to that of Nec-1s.

Finally, Nec-34 was about 10-fold more potent in inhibiting necroptosis of murine cells than that of human cells (Supplementary Fig. S6a). Since Leu159 and Asp180 in hRIPK1 are Val159 and Ser180, respectively, in mRIPK1, we wondered if the counterparts of these two residues in mRIPK1, Val159 and Ser180, respectively, were responsible for stronger inhibition of mRIPK1 by Nec-34. Interestingly, we found that Nec-34 was more potent in inhibiting L159V/D180S hRIPK1 than WT-hRIPK1, providing further validation for our computational model of Nec-34 binding with RIPK1 kinase (Fig. 5g, h). Ser180 may be able to contribute two H-bonds with two nitrogen atoms of Nec-34 at the same time, whereas Asp180 could only contribute one H-bond with the amide nitrogen of Nec-34 (Supplementary Fig. S6b). Overall, these results from mutagenesis studies support our computationally predicted binding mode in the context of live cells. The binding of Nec-34 with RIPK1 kinase may occupy a pocket distinct from all other RIPK1 kinase inhibitors developed to date (Supplementary Fig. S6c).

The sequence of αC-helix and activation loop of RIPK1 differs considerably from those of other RIPKs, and the activation loop of RIPK1 is significant longer than those of RIPK2–4, which could conceivably affect the orientation of the αC-helix interacting with it. Upon alignment of determined co-crystal structures of RIPK1 (PDB 4ITH)^17^ and RIPK2 (PDB 4C8B)^38^, both adopting the “DLG/DFG-out” conformations, the αC helix of RIPK1 is orientated ~30° away from that of RIPK2, allowing the activation loop of RIPK1 to form a hydrophobic pocket that Nec-34 could nicely fit (Supplementary Fig. S6d). Cumulatively, the structural differences of these regions could explain the selectivity of Nec-34 among RIPKs.

### Synergistic inhibitory effect of Nec-1s and Nec-34 on necroptosis

**Fig. 6:**
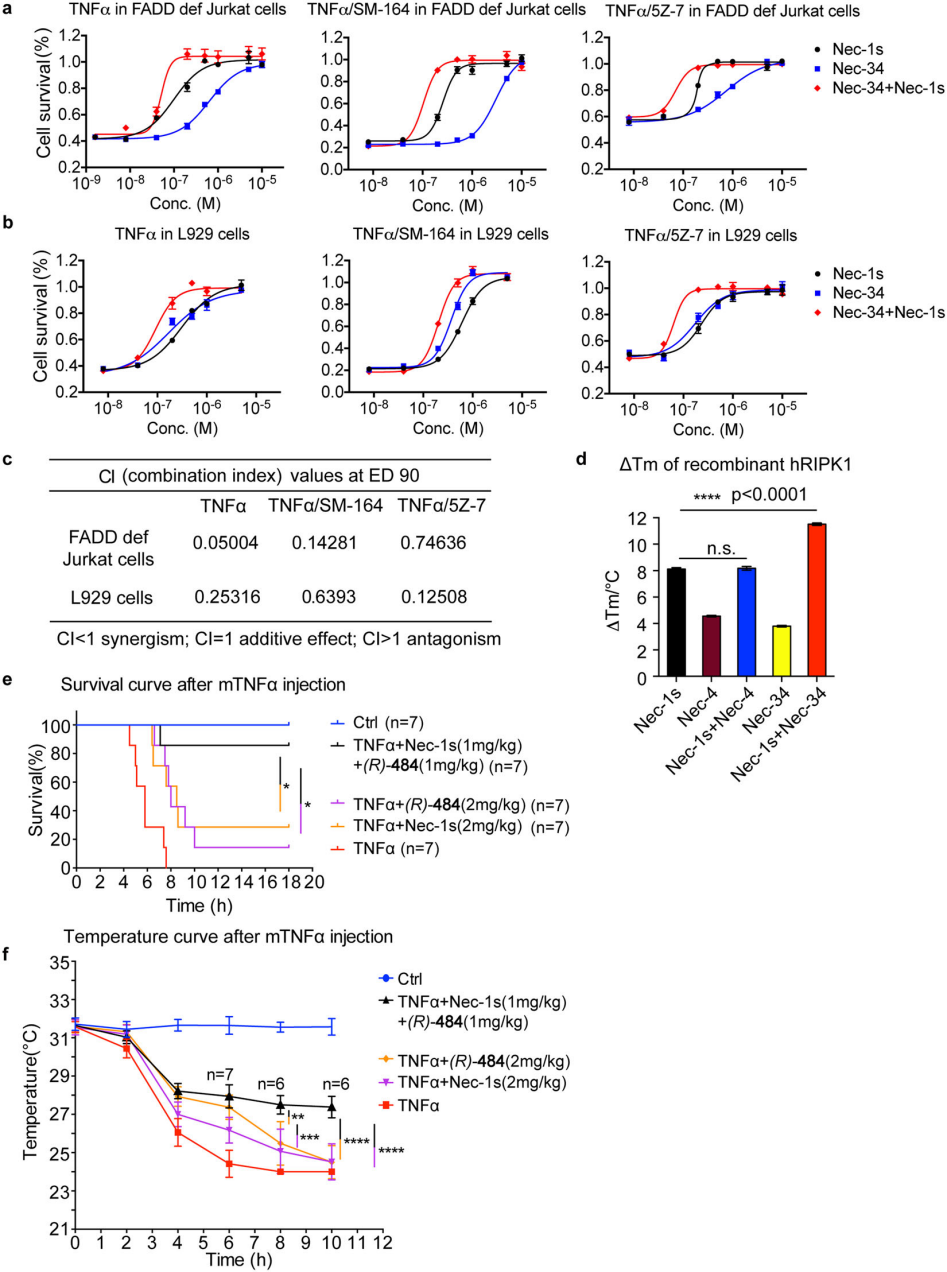
Synergistic inhibitory effects of Nec-1s and Nec-34 on necroptosis. **a**, **b** Synergistic inhibitory effects of Nec-1s and Nec-34 in FADD-deficient Jurkat cells (**a**) and L929 cells (**b**). Cells were pretreated with different concentrations of Nec-1s, Nec-34, or Nec-1s/Nec-34 at a constant ratio 1:1 (e.g., 10 μM Nec-1s alone, 10 μM Nec-34 alone or 5 μM + 5 μM Nec-1s/Nec-34) for 30 min, and then TNFα, TNFα/SM-164 or TNFα/5Z7 were added for an additional 8 h. **c** The combination indexes calculated from **a** and **b**. **d** The Tm values of recombinant hRIPK1 in compounds-treated groups were compared to that of the control group and presented as ΔTm for protein thermal shift assay. The recombinant protein hRIPK1 (residues 1–330, 2 μM) purified from Sf-9 cells was treated with 80 μM Nec-1s, 80 μM Nec-4 and 80 μM Nec-34 alone or with combined Nec-1s/Nec-4 (40 μM + 40 μM) and Nec-1s/Nec-34 (40 μM + 40 μM) for 1 h, protein thermal stability was then analyzed through the differential scanning calorimetry by real-time PCR and the Tm values were calculated by Protein Thermal Shift Software. Three replicates for each reaction were performed. **e**, **f** Synergistic inhibitory effects of Nec-1s and Nec-34 in TNFα-induced SIRS model. Six-week-old C57BL/6J male mice were pretreated intragastrically with Nec-1s (2 mg/kg, *n* = 7), (*R*)-484 (2 mg/kg, *n* = 7) or combination of Nec-1s and (*R*)-484 (1 mg/kg + 1 mg/kg, *n* = 7) for 15 min, and then intravenously injected with mTNFα (0.5 μg per gram mouse body weight, diluted with endotoxin-free PBS). Control mice were injected with vehicle only (endotoxin-free PBS, *n* = 7). Survival period was recorded within 18 h after injection (**e**), and the comparisons of survival curves were performed using Log-rank (Mantel-Cox) test with *P < 0.05. Surface body temperature was recorded within 10 h by an infrared thermometer, and the body temperature of dead mice was set to room temperature (24 °C) (**f**). The cell survival in **a**, **b** was measured by CellTiter-Glo assays. The results shown depict the means ± SEM of *n* = 3 independent biological experiments. *P*-values in **d**, **f** were calculated by two-tailed Student’s *t*-test (n.s., not significant; *P < 0.05, **P < 0.01, ***P < 0.001, ****P < 0.0001).

Given the distinct inhibitory mechanisms of Nec-34 and Nec-1s, we next examined whether the combination of Nec-34 and Nec-1s might synergistically inhibit RIPK1 activation in cells stimulated with TNFα. We characterized the dose-dependent responses of Nec-34 or Nec-1s alone, and the combination of Nec-34/Nec-1s in different concentrations at 1:1 ratio in blocking necroptosis in both human and mouse cell lines (Fig. 6a, b and Supplementary Fig. S7a). The potential cooperative interaction between Nec-34 and Nec-1s was quantitatively assessed using the Chou-Talalay method based on the median-effect equation^39^. This analysis provides a quantitative definition for additive effect (CI = 1), synergism (CI < 1), and antagonism (CI > 1) in drug combinations. As shown in Fig. 6c and Supplementary Fig. S7b, the CI values of Nec-34 and Nec-1s in various cell death models were well below 1 from 0.05 to 0.75, suggesting that Nec-1s and Nec-34 can work in a synergistic manner.

We also determined the synergistic effect of Nec-34 and Nec-1s by using protein thermal shift assay^24^. Combination of Nec-1s and Nec-34, but not Nec-1s and Nec-4, significantly further increased the thermal stability of RIPK1 kinase, compared to the effect of Nec-1s alone (Fig. 6d), which is consistent with the notion that Nec-4 occupies the same binding pocket as that of Nec-1s^10,17^. The synergistic role of Nec-34 and Nec-1s was further confirmed by CETSA, which showed similar effect in co-stabilization of RIPK1 (Supplementary Fig. S7c). Taken together, our results reveal that Nec-1s and Nec-34 exhibit significant synergistic effects in inhibiting RIPK1.

### Compound 484 and Nec-1s cooperate to protect against SIRS in vivo

TNFα-induced systemic inflammatory response syndrome (SIRS) is an animal model of sepsis, which involves both RIPK1-dependent apoptosis and necroptosis^40^. RIPK1 kinase inhibition by genetic modification or small-molecule inhibitors provide protection against lethal septic shock^41,42^. Compound 484 showed improved inhibitory potency against human RIPK1 compared to Nec-34 (Supplementary Table S1). Moreover, compound 484 also provided good oral bio-availability (Supplementary Table S2) and plasma exposures with a moderate half-life of 9 h (Supplementary Table S3) when administered to mice at the concentration of 100 mg compound 484 per kilogram of mouse body weight (mg/kg), which is suitable for in vivo efficacy studies. We further investigated whether the combination of Nec-1s and 484 could exhibit synergistic efficacy in TNFα-induced SIRS model in vivo. The control mice experienced hypothermia and died within 5–8 h after intravenous (i.v.) injection of mTNFα (0.5 μg per gram of mouse body weight, 0.5 μg/g), whereas mice intragastrically pretreated with Nec-1s alone at 15 mg/kg, (*R*)-484 alone at 15 mg/kg or a combination of Nec-1s and (*R*)-484 with each at 7.5 mg/kg were completely protected against the lethality of TNFα-induced SIRS (Supplementary Fig. S7d, e). Reduced doses of either Nec-1s or (*R*)-484 alone at 2 mg/kg provided poor protection against SIRS; whereas the mice treated with a combination of (*R*)-484 and Nec-1s with each at 1 mg/kg showed statistically highly significant protection against lethality and hypothermia induced by SIRS (Fig. 6e, f). Intermediate doses of Nec-1s and (*R*)-484 each at 2.5 mg/kg also provided improved protective effect compared to that of each agent alone at 5 mg/kg (Supplementary Fig. S7f, g). Taken together, we conclude that (*R*)-484 and Nec-1s can also cooperate in protection against TNFα-induced SIRS in vivo.

## Discussion

Inhibitors of RIPK1 kinase have been advanced into human clinical studies for developing new therapies of various devastating human inflammatory and degenerative diseases that still lack effective treatments. For the treatment of chronic human conditions, such as Alzheimer’s disease, Crohn’s disease and rheumatoid arthritis, drug safety is paramount. For any drugs, dose-dependent off-target activities are issues difficult to avoid with long-term dosing. Combining two RIPK1 inhibitors at overall lower dosages might provide an option to achieve an improved efficacy than treatment with single agent, and also possibly reduce the off-target adverse effects specific of either inhibitor alone. Drug combination is already widely applied in treating many diseases such as cancers and virus infections to achieve synergistic therapeutic effects or to avoid the side effects after long-term use of a single agent. Here, we demonstrate a unique mechanism to promote synergistic effects by combining two RIPK1 inhibitors that bind to two different pockets on RIPK1 kinase.

The hydrophobic allosteric pocket of RIPK1 near its catalytic site demonstrates several unique characteristics compared to many other kinases, such as a “DLG motif” instead of “DFG motif” and a “Glu-out” conformation of helix αC upon binding to type-II/III kinase inhibitors^43^. Almost all the reported RIPK1-specific inhibitors target this conserved yet unique hydrophobic pocket that regulates RIPK1 kinase activity, exploiting and stabilizing the “DLG-out”, “Glu-out”, and overall inactive conformations of RIPK1 kinase. In contrast to Nec-1s, which binds to the deep inside part of this hydrophobic pocket, Nec-34 and its analog 484 likely bind in a pocket surrounded by the activation loop, catalytic loop and P + 1 loop. The specifically stabilized peptides in the catalytic loop and activation loop identified by LiP-MS, together with the reduced deuterium uptake of the same loops found in HDX-MS indicate that binding of Nec-34/484 to this pocket may prevent the flexible activation loop from stretching out and maintain RIPK1 in “DLG-out” inactive conformation. Consistently, the abundance of 154-IADLGLASFKMW-165 peptide, where “DLG” is located, was increased in both Nec-1s- and Nec-34-treated RIPK1 kinase in LiP-MS analysis. Thus, Nec-34/484 and Nec-1s may be able to keep RIPK1 kinase in the same “DLG-out” inactive conformation. Since our model predicts that Nec-34 may bind to the activation loop opposite to where Nec-1s binds, it is reasonable to predict that the binding of Nec-1s and Nec-34 can stabilize the “DLG-out” inactive conformation like fixing two “nails” on the opposite sides of the activation loop. Hence, the discovery of this binding pocket defined by Nec-34 not only reveals a new regulatory region of RIPK1 kinase, but also provides an opportunity to achieve more efficient inhibition of RIPK1 and necroptosis by exploiting the synergistic cooperation of Nec-34/484 and Nec-1s.

The synergistic effects of Nec-34 with Nec-1s suggest that the conformation of RIPK1 stabilized by single agent may remain a certain level of flexibility, so that the co-occupation of the pockets by both Nec-34 and Nec-1s may lead to more effective and stable inhibition of the kinase, compared with the effect of either inhibitor alone. The combination of the unusual dynamics of RIPK1 and the unique binding mode of necrostatins may provide additional levels of selectivity for future rational design of small-molecule inhibitors of RIPK1 kinase.

## Materials and methods

### Reagents and antibodies

The following commercial antibodies and reagents were used in this study: RIPK1 (Cell Signaling Technology, #3493); p-S166 RIPK1 (Cell Signaling Technology, #31122); p-RIPK3 (Cell Signaling Technology, #91702, mouse specific); p-RIPK3 (Cell Signaling Technology, #93654, human specific); FADD (Abcam, #ab124812; Santa Cruz, #6036); TRADD (Santa Cruz, #46653); α-Tubulin (Sigma-Aldrich, #T9026); β-actin (Santa Cruz, #81178); Flag (Cell Signaling Technology, #2368); Flag-TNFα (Enzo, Life Sciences); Flag antibody-conjugated agarose (Sigma, #A2220); Mouse TNFα (Cell Sciences); p-S345 MLKL (Abcam, #ab196436); IκBα (Cell Signaling Technology, #4814); p-IκBα (Cell Signaling Technology, #2859); IKKα (Cell Signaling Technology, #2682); IKKβ (Cell Signaling Technology, #8943); p-IKKα/β (Cell Signaling Technology, #2697); p38 (Cell Signaling Technology, #8690); p-p38 (Cell Signaling Technology, #9216); JNK (Cell Signaling Technology, #9252); p-JNK (Cell Signaling Technology, #4668); p65 (Cell Signaling Technology, #8242); p-p65 (Cell Signaling Technology, #3033); MK2 (Cell Signaling Technology, #3042); p-MK2 (Cell Signaling Technology, #3041); caspase-8 (Cell Signaling Technology, #4927); SYRO Orange Protein gel stain (Life Technologies, #s6651); 5Z7 (Sigma-Aldrich, #O9890); dimerizing agents AP20187 (MCE, #HY-13992); Sf-9 cell medium ESF-921 (ExpressionSystems, #96-001-01); ADP-Glo™ kinase assay (Promega, #V6930).

### Cell culture

HEK293T, MEFs, HeLa, H4, L929 and NIH/3T3 cells were cultured in DMEM (Gibco) with 10% (vol/vol) FBS (Gibco) and 100 units/mL penicillin/streptomycin. Jurkat cells were cultured in RPMI-1640 (Gibco) with 10% (vol/vol) FBS (Gibco) and 100 units/mL penicillin/streptomycin. HT29 cells were cultured in McCoy’s 5A medium (Gibco) supplemented with 10% (vol/vol) FBS (Gibco) and 100 units/mL penicillin/streptomycin. All cells were cultured in 37 °C with 5% CO_2_. Sf-9 insect cells were cultured in ESF-921 medium at 27 °C with a shaking rate of 120 rpm.

### High-throughput screen (HTS) for inhibitors of necroptosis

The primary HTS was performed using the small molecule libraries at 10 μM on FADD-deficient Jurkat cells treated with TNFα and L929 cells treated with zVAD.fmk in 384-well plate in University of Pittsburgh Molecular Libraries Screening Center (PMLSC). Hits exhibiting > 75% inhibition of cell death in the primary screen were cherry picked and tested in two independent confirmation runs. Reproducible active hits were further subjected to 10-points twofold dilution series concentration response assay starting from a maximum concentration of 20 μM. Data of the primary and confirmatory screen and detail procedures have been deposited in PubChem BioAssay database and are accessible through the links below:

AID# 1377 (https://pubchem.ncbi.nlm.nih.gov/bioassay/1377),

AID# 463075 (https://pubchem.ncbi.nlm.nih.gov/bioassay/463075),

AID# 463117 (https://pubchem.ncbi.nlm.nih.gov/bioassay/463117),

and AID# 463178 (https://pubchem.ncbi.nlm.nih.gov/bioassay/463178).

The detailed information of active compounds, including chemical structures, chemical and physical properties, biological activities, safety and toxicity information, etc., are inter-linked to database of PubChem Compound. The record of Nec-34 can be tracked by its CID# using the following link: https://pubchem.ncbi.nlm.nih.gov/compound/3841655.

### Cell viability assays

General cell survival was measured by the ATP luminescence assay CellTiterGlo (Promega). The percentage of viability was normalized to readouts of vehicle-treated cells of each genotype.

### Immunoprecipitation

Cells were lysed with Nonidet P-40 buffer (25 mM Tris HCl at pH 7.4, 150 mM NaCl, 1 mM EDTA, 1 mM EGTA, 1% Nonidet P-40, 10% glycerol) supplemented with 1 mM PMSF, 1× protease inhibitor cocktail (Selleckchem), 10 mM β-glycerophosphate, 5 mM NaF, and 1 mM Na_3_VO_4_. Debris was precipitated, and the supernatant was incubated with appropriate antibody overnight at 4 °C. The immunocomplex was captured by protein A/G agarose (Life Technologies) for additional 1–2 h at 4 °C. Beads were washed four times, and the immunocomplex was eluted from beads by 2× laemmli sample buffer.

### Protein purification

For the purification of RIPK1 from 293T cells, Flag-tagged FL-RIPK1 or Flag-tagged truncated RIPK1 (residues 1–330) were transfected to 293T cells for 24 h. Cells were then lysed with Nonidet P-40 buffer supplemented 1× protease inhibitor cocktail (Selleckchem). Debris was precipitated, and the supernatant was incubated with anti-Flag M2 affinity agarose gel overnight at 4 °C. Beads were washed four times and equally divided into several parts for in vitro kinase assay.

For the purification of RIPK1 from Sf-9 cells, we thank Professor Yigong Shi for providing the construct of RIPK1 (residues 1–294) with four cysteine residues (C34/127/233/240) mutated to alanine^17^. The WT or four cysteine residues-mutated form of *RIPK1* (residues 1–330) were subcloned into the same *Nde*I and *Xho*I sites to replace RIPK1 (residues 1–294). Bacmids were generated in DH10Bac cells, and the resulting baculoviruses were generated by three rounds of amplification in Sf-9 insect cells. After infection by baculoviruses for 48 h, the cells were harvested through centrifugation, and the pellets were resuspended in lysis buffer (25 mM Tris, 150 mM NaCl, pH 8.0). Cells were then homogenized with an ultra-high-pressure cell disrupter at 4 °C and lysate was collected through centrifugation at 12,000 rpm. The recombinant protein was further purified by nickel affinity chromatography (GE Healthcare), anion-exchange chromatography (Source-15Q, GE Healthcare), and gel-filtration chromatography (Superdex-200, GE Healthcare). The purified RIPK1 was stored in a buffer containing 25 mM Tris (pH 8.0), 150 mM NaCl, and 5 mM dithiothreitol.

### Thermal shift assay

Recombinant hRIPK1 (residues 1–330, 2 μM) was mixed with the SYRO Orange Protein dye (final concentration 10×) in a final reaction volume of 10 μL. The protein thermal stability was analyzed by differential scanning calorimetry on a QuantStudio™ 7 Flex Real-Time PCR System according to the manufacturer’s recommendations using a melt protocol of 25 to 95 °C at a 1% ramp rate (equivalent to 0.015 °C/s). Data were analyzed using the Protein Thermal Shift™ Software v1.3.

### Cellular thermal shift assay

The procedure was modified from the reported procedure by Molina^25^. MEFs were treated with Nec-1s or Nec-34 at 10 μM for 2 h, and then harvested and resuspended in 1/10 volume of pre-warmed PBS containing protease inhibitor cocktail. The cell suspensions were then divided into Eppendorf tubes (100 μL/tube) and transiently heated to different temperatures ranging from 46 to 56 °C for 3 min using thermal-mixer. The heat-treated cell suspensions were freeze-thawed three times using liquid nitrogen, and centrifuged at 20,000× *g* for 10 min at 4 °C to separate the soluble proteins from the cell debris and aggregates. The supernatant containing the remaining soluble proteins was transferred to new tubes and analyzed by sodium dodecyl sulphate–polyacrylamide gel electrophoresis followed by western blotting.

### ADP-Glo™ kinase assay

Recombinant hRIPK1 (residues 1–330, 1 μM) were pretreated with compounds for 30 min. The kinase reactions were initiated by ATP in 1× in vitro kinase assay buffer containing 50 mM HEPES, 50 mM NaCl, 30 mM MgCl_2_, 1 mM DTT, 0.05% BSA, 0.02% CHAPS, and the reactions were carried out at 25 °C for 2 h. After the kinase reaction, the assay was performed in two steps: (1) ADP-Glo™ Reagent was added to terminate the kinase reaction and deplete the remaining ATP, and (2) the Kinase Detection Reagent was added to convert ADP to ATP and allowed the newly synthesized ATP to be measured using a luciferase reaction.

### Photo-affinity labeling and click chemistry

Cells were pretreated with 20 μM photo-affinity probe 496 or photostable control compound 484 for 1 h, and then lysates were prepared at a concentration of 2 mg/mL total protein in Nonidet P-40 buffer. The whole-cell lysates, or the purified hRIPK1 (residues 1–330) proteins were individually treated with 200 μM 484 or 496 for 30 min, and all samples were photo-crosslinked at ∼350 nm on ice for 30 min using a UV crosslinker (energy: 1200). After photo-affinity labeling, click chemistry was preformed to allow the linkage of Biotin-PEG3-Azide to enable pulldown^26^. A master mix of the catalyst (final concentration) was prepared immediately before use by combining: 100 μM Biotin-PEG3-Azide (TCI (Shanghai) Development Co.,Ltd.), 100 μM TBTA (TCI (Shanghai) Development Co.,Ltd.), 1 mM CuSO_4_ (Sinopharm Chemical Reagent Co.,Ltd.) 1 mM CuBr (Shanghai Macklin Biochemical Technology Co.,Ltd.), 1 mM TCEP (Sun Chemical Technology (Shanghai) Co.,Ltd.). The samples were vortexed and incubated for 1 h at room temperature and TCEP was added one more time after incubation for 30 min. Chloroform-methanol precipitation was preformed next to isolate proteins. The biotin-labeled proteins were isolated by incubating with streptavidin-coupled beads overnight at 4 °C.

### Photo-crosslinking-coupled mass spectrometry

The recombinant hRPK1 protein was pulled down using streptavidin beads, and then digested by trypsin on beads. After washing out free peptides, the biotinylated peptides were eluted from beads with 30% Acetonitrile and 2% formic acid. The resulting peptides were analyzed on a Q Exactive HF mass spectrometer (Thermo Scientific) in a data-dependent mode. The MS/MS data were subjected to the database search against a UniProt human protein database in Proteome Discoverer 1.4 (Thermo Scientific). The precursor mass tolerance was set as 10 ppm, and the fragment mass tolerance was set as 0.1 Da. The cysteine carbamidomethylation was set as a static modification. The mass shift of 918.2587 Da was set as a variable modification on four or five amino acids in every round database search until all 20 proteinogeic amino acids were covered. The FDR at peptide spectrum match level was controlled below 1%.

### GaMD enhanced sampling simulations and clustering of compound 484

To predict the potential binding site of compound 484 on RIPK1 surface, we first selected 50 equally distributed sites with a distance of 15 Å away from the protein surface, and then placed a single 484 molecule at each site to obtain a total of 50 initial complexes. Only one ligand was used per complex. The GaMD enhanced sampling simulations were applied to all initial complexes to obtain possible binding sites and corresponding binding pose. In preparation for molecular dynamics simulations, the RIPK1 structure was obtained from RCSB PDB dataset (ID 6C4D) and the missing activation loop atoms were built with Modeler. The electrostatic potential of 484 was calculated in Gaussian09 (Gaussian, Inc., Wallingford CT, USA) program with HF/6–31G* basis set and was used to derive RESP partial charges. The Amber99sb force field was used for the protein and general Amber force field (GAFF) for the ligand.

All simulations of initial complexes were carried out using Amber 16 program (University of California, San Francisco, USA). For each initial complex, the structure was immersed in a truncated octahedron solvent box of TIP3P water with at least 10 Å distance between the complex surface and the box edge, followed by the addition of 0.15 M NaCl to neutralize the system. Energy minimization was then performed with a gradually reduced position restraint on the complex heavy atoms to remove overlapping coordinates in the system. Next, the minimized system was subjected to 500 ps constant volume (NVT) and 500 ps constant pressure (NPT) equilibration with weaker position restraints on the complex heavy atoms to further relax the system. During the NPT runs, all bonds involving hydrogen were constrained using the SHAKE algorithm to enable an integration time step of 2 fs. A cutoff of 1.0 nm was set for non-bonded interactions and the electrostatic interaction beyond that was treated with particle mesh Ewald (PME) method. The Langevin Thermostat was applied to maintain the temperature to 300 K, and Berendsen Barostat was used to keep the pressure at 1.0 bar. All simulation trajectories were saved at an interval of 10 ps.

The subsequent GaMD protocol consisted of three steps: equilibration simulation with no boost potential (NPT ensemble, 12 ns), GaMD simulation with updated boost potential (NPT ensemble, 28 ns), and final production simulation with fixed acceleration parameters (NPT ensemble, 200 ns). The boost potential was applied to both (i) the torsional terms only and (ii) across the entire potential to facilitate the exploring of entire conformational space. To further extend the sampling space, five independent production simulations with random initial velocities were performed for each complex system, achieving a total of 50 μs trajectories. We collected 5 × 10^6^ coordinates from all production runs and carried out analysis based on these snapshots. With all GaMD-generated snapshots, the location of ligand-binding site could be inferred from the probability of each RIPK1 residue in contact with the ligand. A contact is defined if the shortest heavy atom distance between the residue and ligand is smaller than 4.5 Å. Generally, residues with higher contact probability show higher possibility to constitute the ligand-binding site, and the representative binding pose can be extracted by further structure clustering of the ligands located within this site. Before the structure clustering analysis, all snapshots should be superimposed onto a reference structure using corresponding protein conformations. The residue−ligand contacts were calculated by our in-house scripts and the ligand structure clustering was performed using the CPPTRAJ module of Amber 16 program.

### LiP-MS

Recombinant hRIPK1 (residues 1–330, 20 μg) were treated with vehicle (0.4% DMSO), Nec-1s (200 μM) and Nec-34 (200 μM), respectively, for 2 h at room temperature. Samples were then digested with proteinase K at 1:100 (proteinase K:protein, w/w) for 3 min at 25 °C, and incubated at 98 °C for 3 min to inactivate proteinase K. Denaturation was performed by addition of 10% sodium deoxycholate at 1:1 ratio. Samples were further digested with Trypsin (Promega) at 1:100 (Trypsin:protein, w/w) overnight at 37 °C. Peptide mixtures were desalted with mono-spin C18 column (GL Sciences). Equal amount peptide of each sample was loaded onto a homemade 30 cm-long pulled-tip analytical column (ReproSil-Pur C18 AQ 1.9 μm particle size, Dr. Maisch GmbH, 75 μm ID × 360 μm OD) connected to an Easy-nLC1200 UHPLC (Thermo Scientific) for mass spectrometry analysis.

The acquired MS/MS data were analyzed against a reconstructed database (UniProtKB Spodoptera frugiperda database (database released on Jul 1st, 2020) with recombinant hRIPK1 sequence) by Andromeda algorithm built-in MaxQuant engine (v1.6). Trypsin was defined as cleavage enzyme. A decoy database containing the reversed sequences of all the proteins was appended to the target database to accurately estimate peptide probabilities and false discovery rate (FDR), and FDR was set at 0.01. Linear fit model was used to fit the intensity of every peptide across all the samples for sake of good performance at filtering out significantly changed peptides. The investigation aims at identifying conformotypic peptides that significantly change their abundance between the control and compounds-treated groups. Each condition was tested with three biological replicates and statistically tested for differential conformotypic peptide abundances between conditions applying a logarithmic fold-change cutoff of 1 and adjusted *P*-value cutoff of 0.05. Peptide abundance statistics are obtained by grouping different precursor ions of the same peptide sequences.

### HDX-MS

Recombinant hRIPK1 (residues 1–330, 100 μg) were treated with vehicle (0.4% DMSO) and Nec-34 (200 μM), respectively for 2 h at room temperature. Samples were processed automatically by a LEAP Technologies Hydrogen Deuterium Exchange PAL system (Carrboro, NC). HDX measurements were taken at 0 s, 30 s, 100 s, 300 s, 1000 s, and 3000 s at 4 °C. After each time point, an aliquot of sample was transferred to a vial in a 0.5 °C chamber and quenched by addition of an equal volume of quench buffer (200 mM citric acid, 4 M guanidine-HCl, 500 mM TCEP in H_2_O, pH 2.3) for 0.5 min prior to online digestion. The complete HDX-MS procedure was repeated three times for each sample and each time point. Online digestion, trapping, desalting process and separation process was then performed in the temperature-controlled compartment of the HDX PAL system. Data were acquired using a Thermo LTQ Orbitrap-Elite mass spectrometer (San Jose, CA) with a Thermo H-ESI II probe. For peptide identification, mass spectra were acquired in a data-dependent scan using FTMS mode in MS1 (one microscan, 100 ms max injection time, 60 k resolution at 400 *m*/*z*) at the *m*/*z* range of 300–1500 followed by ten CID MS2 scans in the ion trap with *a* ± 2.0 *m*/*z* isolation width. Once the peptides were identified, the deuterium uptake in HDX experiments was conducted using FTMS mode in MS1.

The spectra generated were searched in PEAKS Studio X against an homemade database including all target proteins with a precursor mass tolerance of ≤ 20 ppm and MS/MS fragment ≤ 0.02 Da. Retention time and sequence information for each peptide were exported to Excel for HDX data processing. HDX data analysis was carried out using HDExaminer 2.0 (Sierra Analytics Inc., Modesto, CA). The number of deuterium taken up by each peptide at each exchange time was calculated by the software algorithm for matching the best theoretical isotope distribution pattern to the observed isotope distribution pattern. Deuterium uptake was plotted as a function of exchange time. Triplicate runs were compared using Student’s *t*-test at the 95% confidence level to confirm the consistency of the analytical results obtained. Deuterium uptake was converted to %D for each peptide based on the theoretical number of D; %D was used to generate heat maps. In addition, H/D-ex analysis was also carried out on non-deuterated and fully deuterated samples to correct back-exchange.

### TNFα-induced SIRS model

Six-week-old C57BL/6J male mice were pretreated intragastrically with Nec-1s, (*R*)-484 or combination of Nec-1s/(*R*)-484 at indicated concentrations (in a volume of 200 µL per 20 g mouse) for 15 min before i.v. injection of mTNFα (in a dose of 0.5 μg per gram of mouse body weight, diluted in endotoxin-free PBS). Control mice were injected with vehicle only (endotoxin-free PBS, in a volume of 5 µL per gram of mouse body weight). Surface body temperature was recorded during 10 h with an infrared thermometer. Survival was recorded during 18 h and mice were promptly sacrificed when body temperature was below 24 °C (room temperature). The body temperature of dead mice was set to 24 °C (room temperature). Mice used were randomly divided into different groups and experiments were conducted by G.W. and X.Z. in double-blinded manner. G.W. performed the intragastrical treatment of compounds and i.v. injection of mTNFα. X.Z. recoded the temperature and survival of mice.

Nec-1s and (*R*)-484 were dissolved in different formulations to ensure the best solubilities of these drugs. Nec-1s was dissolved in 0.5% CMC with a final concentration of 3 mg/mL. (*R*)-484 was dissolved in ethanol and then triple amount of copovidine was added (Star-Tech & JRS Specialty Products). The mixture was stirred for 30 min to be clear and ethanol was removed under reduced pressure and high vacuum for 2 h. The mixture was further dissolved in 0.5% HPMC with a final concentration of 3 mg/mL until a homogeneous suspension was formed. To remove the potential experimental bias of different formulations, 3 mg/mL Nec-1s was further diluted in copovidine containing 0.5% HPMC to a final concentration of 1.5 mg/mL and delivered intragastrically in a volume of 200 µL per 20 g mouse, 15 mg/kg. 3 mg/mL (*R*)-484 was diluted in 0.5% CMC to a final concentration of 1.5 mg/mL and delivered intragastrically in a volume of 200 µL per 20 g mouse, 15 mg/kg. Equal amount of 3 mg/mL Nec-1s and 3 mg/mL (*R*)-484 were mixed to obtain 1.5 mg/mL combination of Nec-1s/(*R*)-484 and the mixture was delivered intragastrically in a volume of 200 µL per 20 g mouse, 15 mg/kg. Compound solutions with concentrations of 0.5 mg/mL (delivered intragastrically in a volume of 200 µL per 20 g mouse, 5 mg/kg) and 0.2 mg/mL (delivered intragastrically in a volume of 200 µL per 20 g mouse, 2 mg/kg) were diluted from 1.5 mg/mL stock with mixed 0.5% CMC and copovidine containing 0.5% HPMC.

## Supplementary information

supplementary information
