# Supplementary material for: Discovery of a cooperative mode of inhibiting RIPK1 kinase
Source: Cell Discov. 2021 Jun 1;7:41. doi: 10.1038/s41421-021-00278-x (PMC8169668; doi:10.1038/s41421-021-00278-x)
Supplement: Supplementary file 1 — supplementary information [file 41421_2021_278_MOESM1_ESM.pdf]

Supplementary information

Chemical synthesis methods of Nec-34 and its derivatives.

General method. All chemical reagents and solvents were obtained from commercial sources and used without further purification. Anhydrous solvents were purchased from J&K Scientific, Beijing, China. ^{1}H NMR and ^{19}F NMR spectra were acquired on Bruker Ultrashield TM 400Plus spectrometer. All compounds were determined to be >95% pure by LC-MS using an Agilent Technologies 6120 Quadrupole LC-MS concurrently with UV chromatography and an Agilent Poroshell 120 EC-C18 column (2.7 μm, 3.0 x 50 mm) eluted with a gradient of 5-95% of acetonitrile in water (containing 0.1% formic acid) over 6 min at a flow rate of 0.5 mL/min at 40 °C. Prep-HPLC was carried on Agilent Technologies 1260 Infinity and XTerra Prep MS C18 column (s-10 μm, 19 x 250 mm) eluted with 50% of acetionitrile in water (containing 0.1% formic acid). Chiral resolution was performed on Agilent Technologies 1260 Infinity and DAICEL CHEMICAL INDUSTRIES LTD CHIRALCEL OJ column (s-10 μm, 4.6 x 250 mm) eluted with a mixture of EtOH/n-Hexane. X-ray was performed on D8 Venture TXS PHOTON II with liquid N_2 as coolant. It is noteworthy that the **Nec-34** and its derivatives such as **484** and **496** were used in all tests as racemates unless specified otherwise.

Scheme 1. Synthesis of **Nec-34**[a]

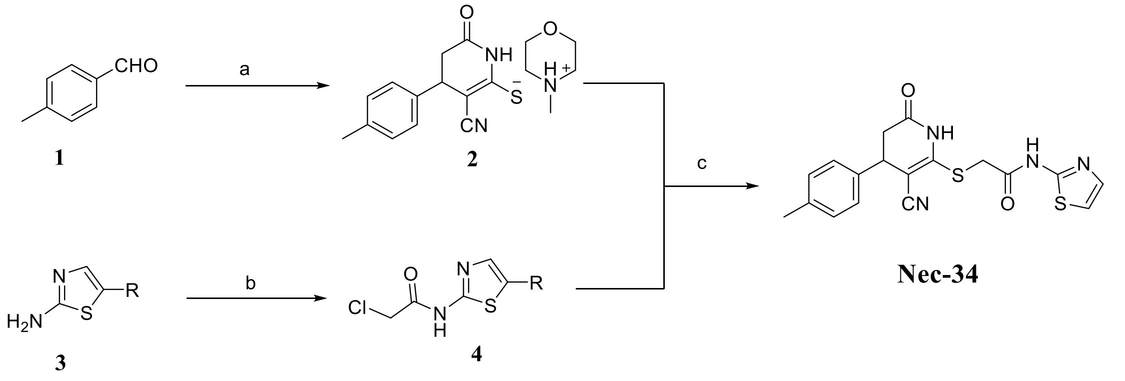

Reagents and conditions: (a) 2-cyanothioacetamide, 4-methylmorpholine, Meldrum's acid, EtOH, r.t.- reflux, 5 h; (b) chloroacetyl chloride, Et_3N, DCM, 0 °C, 1.5 h; (c) tetrabutylammonium iodide, DMA, r.t., 10 min.

Step 1 (*R*/*S*)-2-mercapto-6-oxo-4-(*p*-tolyl)-1,4,5,6-tetrahydropyridine-3-carbonitrile, 4-methylmorpholin-4-ium salt (**2**).

A solution of *p*-tolualdehyde (2.40 g, 20 mmol), 2-cyanothioacetamide (2.00 g, 20 mmol) and 4-methylmorpholine (0.50 mL) in EtOH (30 mL) was stirred at r.t. for 1 hour. Meldrum's acid (2.99 g, 20.8 mmol) and 4-methylmorpholine (2.80 mL) were then added to the mixture. After stirring for 1 h at r.t., the reaction mixture was heated to reflux under N_2 for 4 h. The reaction mixture was cooled to r.t. and the solid was collected by filtration, washed with cold EtOH (15 mL) and air-dried to afford compound **2** (1.20 g, 17.4%) as a yellow solid. ^{1}H NMR (400 MHz, DMSO-d_6) δ 9.66 (br, 1H), 8.58 (s, 1H), 7.09 (d, J = 8.4 Hz, 2H), 7.05 (d, J = 8.4 Hz, 2H), 3.76 (bs, 4H), 3.59 (dd, J = 7.1, 4.4 Hz, 1H), 3.32 (bs, 3H), 2.76 (s, 3H), 2.70 (dd, J = 16.0, 7.2 Hz, 1H), 2.31 (dd, J = 16.0, 4.4 Hz, 1H), 2.26 (s, 3H). LC-MS (ESI, +ve) *m/z*: $[M + H]^+$ calcd for $C_{13}H_{12}N_2OS$ 244.07; found, 245.1.

Step 2 2-chloro-*N*-(thiazol-2-yl)acetamide (**4**).

To a solution of 2-aminothiazole **3** (1.00 g, 10 mmol) and Et_3N (2.02 g, 20 mmol) in DCM (20 mL) stirred at 0 °C was added chloroacetyl chloride (1.23 g, 11 mmol) dropwise. After stirring for 1.5 h, water (30 mL) was added to the mixture, and the organic phase was separated and concentrated. The residue was washed with PE (30 mL) and dried in vacuo to afford compound **4** (1.62 g, 92%) as a brown solid. ^1H NMR (400 MHz, DMSO-d_6) δ 12.45 (s, 1H), 7.51 (d, J = 3.6 Hz, 1H), 7.29 (d, J = 3.6 Hz, 1H), 4.39 (s, 2H). LC-MS (ESI, +ve) *m/z*: $[M + H]^+$ calcd for $C_5H_5ClN_2OS$ 175.98 and 177.98; found, 177.0 and 179.0 .

Step 3 (*R*/*S*)-2-((3-cyano-6-oxo-4-(*p*-tolyl)-1,4,5,6-tetrahydropyridin-2-yl)thio)-*N*-(thiazol-2-yl)acetamide (**Nec-34**).

A solution of **2** (3.25 g, 9.41 mmol), **4** (1.50 g, 8.47 mmol) and tetrabutylammonium iodide (0.35 g, 0.94 mmol) in DMA (30 mL) was stirred at r.t. for 10 min. The reaction mixture was then poured into water (150 mL) slowly. The solid was collected by filtration, washed with water (100 mL), $CHCl_3$ (100 mL) and dried in vacuo afford **Nec-34** (2.50 g, 70%) as a white solid. ^1H NMR (400 MHz, DMSO-d_6) δ 12.45 (s, 1H), 10.64 (s, 1H), 7.51 (d, J = 3.6 Hz, 1H), 7.29 (d, J = 3.6 Hz, 1H), 7.15 (d, J = 8.1 Hz, 2H), 7.10 (d, J = 8.1 Hz, 2H), 4.07 (s, 2H), 3.99 (t, J = 6.4 Hz, 1H), 2.89 (dd, J = 16.3, 7.3 Hz, 1H), 2.57 (dd, J = 16.4, 5.8 Hz, 1H), 2.27 (s, 3H). LC-MS (ESI, +ve) *m/z*: $[M + H]^+$ calcd for $C_{18}H_{16}N_4O_2S_2$ 384.07; found, 385.0 .

Scheme 2. Synthesis of **484**[a]

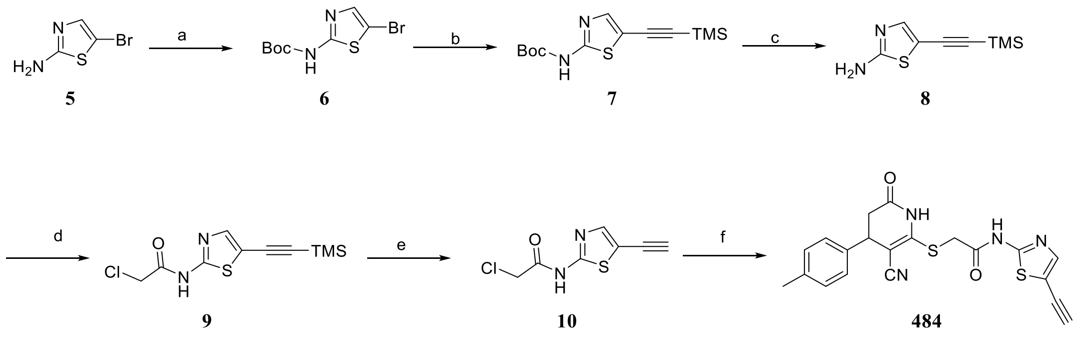

Reagents and conditions:(a) $(boc)_2O$, DMAP, THF, r.t., 5 h; (b) trimethylethynysilene, $Pd(PPh_3)_2Cl_2$, CuI, Et_3N, 65 °C, 4 h; (c) TMSOTf, 2,6-lutidine, DCM, 0 °C- r.t., 4.5 h; (d) chloroacetyl chloride, Et_3N, DCM, 0 °C, 1.5 h; (e)K_2CO_3, MeOH, r.t., 2.5 h; (f) **2**, tetrabutylammonium iodide, DMA, r.t., 2 h.

Step 1 *tert*-butyl (5-bromothiazol-2-yl)carbamate (**6**).

A solution of 5-bromothiazol-2-amine **5** (20.00 g, 112.30 mmol) , DMAP (0.69 g, 5.62 mmol) and $(Boc)_2O$ (25.72 g, 117.97 mmol) in THF (300 mL) was stirred at r.t. for 5 h. The reaction mixture was then concentrated under reduced pressure. The residue was dissolved in EtOAc (300 mL) and the solution was filtered and concentrated under reduced pressure. The residue was suspended in PE (50 mL) and PE was decanted to afford the title compound **6** (30 g, 96.2%) as a yellow solid. ^1H NMR (400 MHz, $CDCl_3$) δ 10.86 (s, 1H), 7.24 (s, 1H), 1.57 (s, 9H). LC-MS (ESI, +ve) *m/z*: $[M + H]^+$ calcd for $C_8H_{11}BrN_2O_2S$ 277.97 and 279.97; found, 222.9 and 224.9 (the *tert*-butyl was lost under acidic condition of LC-MS).

Step 2 *tert*-butyl (5-((trimethylsilyl)ethynyl)thiazol-2-yl)carbamate (**7**)

To a solution of *tert*-butyl (5-bromothiazol-2-yl)carbamate **6** (8.00 g, 28.77 mmol), $Pd(PPh_3)_2Cl_2$ (3.00 g, 4.32 mmol) and CuI (1.00 g, 5.75 mmol) in Et_3N (8 mL) and THF (80 mL) was added trimethylethynysilene (4.23 g, 43.16 mmol) under N_2 atmosphere. After stirring

at 65 °C for 4 h, the reaction mixture was concentrated under reduced pressure, and the residue was dissolved in EtOAc (100 mL) and the solution was washed with water (2 x 100 mL). The organic phase was dried over anhydrous Na_2SO_4, concentrated and the residue was purified by silica gel column chromatography (PE : EtOAc = 10 : 1) to afford the title compound **7** (6.2 g, 73.0%) as a yellow solid. ^{1}H NMR (400 MHz, $CDCl_3$) δ 11.07 (s, 1H), 7.45 (s, 1H), 1.57 (s, 9H), 0.24 (s, 9H). LC-MS (ESI, +ve) *m/z*: $[M + H]^+$ calcd for $C_{13}H_{20}N_2O_2SSi$ 296.10; found, 297.1.

Step 3 5-((trimethylsilyl)ethynyl)thiazol-2-amine (**8**).

To a solution of *tert*-butyl (5-((trimethylsilyl)ethynyl)thiazol-2-yl)carbamate (20.00 g, 67.56 mmol) and 2,6-lutidine (14.48 g, 135.10 mmol) in DCM (200 mL) cooled at 0 °C was added TMSOTf (22.52 g, 101.30 mmol) dropwise. After stirring at r.t. for 4.5 h, the reaction mixture was washed with water (2 x 150 mL). The DCM phase was dried over anhydrous Na_2SO_4, concentrated and the residue was purified by silica gel column chromatography (PE : EtOAc = 8 : 2) to afford the title compound **8** (10.40 g, 78.5%) as a yellow solid. ^{1}H NMR (400 MHz, $CDCl_3$) δ 7.24 (s, 1H), 5.08 (s, 2H), 0.22 (s, 9H). LC-MS (ESI, +ve) *m/z*: $[M + H]^+$ calcd for $C_8H_{12}N_2SSi$ 196.05; found, 197.1.

Step 4 2-chloro-*N*-(5-((trimethylsilyl)ethynyl)thiazol-2-yl)acetamide (**9**).

To a solution of 5-((trimethylsilyl)ethynyl)thiazol-2-amine **8** (10.40 g, 53.06 mmol) and Et_3N (10.71 g, 106.12 mmol) in DCM (100 mL) cooled at 0 °C was added chloroacetyl chloride (7.13 g, 63.67 mmol) dropwise. After stirring for 1.5 h, the reaction was quenched with water (150 mL) and the organic layer was separated, concentrated. The residue was suspended in PE (100 mL) and PE was decanted to afford the title compound **9** (10.6 g, 73.5%) as a yellow solid.

^1H NMR (400 MHz, $CDCl_3$) δ 9.93 (s, 1H), 7.60 (s, 1H), 4.28 (s, 2H), 0.25 (s, 9H). LC-MS (ESI, +ve) *m/z*: [M + H]$^+$ calcd for $C_{10}H_{13}ClN_2OSSi$ 272.02 and 274.02; found, 273.0 and 275.0.

Step 5 2-chloro-*N*-(5-ethynylthiazol-2-yl)acetamide (**10**).

A solution of 2-chloro-*N*-(5-((trimethylsilyl)ethynyl)thiazol-2-yl)acetamide **9** (1.00 g, 3.68 mmol) and K_2CO_3 (0.56 g, 4.04 mmol) in MeOH (12 mL) was stirred at r.t. for 2.5 h. The reaction mixture was then concentrated to afford the title compound **10** which was used for next step without further purification. ^1H NMR (400 MHz, DMSO-d_6) δ 12.81 (s, 1H), 7.79 (s, 1H), 4.63 (s, 1H), 4.41 (s, 2H). LC-MS (ESI, +ve) *m/z*: [M + H]$^+$ calcd for $C_7H_5ClN_2OS$ 199.98 and 201.98; found, 201.0 and 203.0.

Step 6 (*R*/*S*)-2-((3-cyano-6-oxo-4-(p-tolyl)-1,4,5,6-tetrahydropyridin-2-yl)thio)-*N*-(5-ethynylthiazol-2-yl)acetamide (**484**).

A solution of **2** (3.60 g, 10.58 mmol), 2-chloro-*N*-(5-ethynylthiazol-2-yl)acetamide **10** (2.11 g, 10.58 mmol) and tetrabutylammonium iodide (0.39 g, 1.06 mmol) in DMA (15 mL) was stirred at r.t. for 2 h. The reaction was then quenched with water (150 mL) and the reaction mixture was extracted with EtOAc (2 x 100 mL). The combined organic phase was dried over anhydrous Na_2SO_4, concentrated and the residue was purified by silica gel column chromatography (DCM : EtOAc = 6 : 4) to obtain **484** as a yellow solid (4.00 g, 94%). ^1H NMR (400 MHz, DMSO-d_6): δ 12.78 (s, 1H), 10.65 (s, 1H), 7.79 (s, 1H), 7.14 (d, *J* = 8.0 Hz, 2H), 7.09 (d, *J* = 8.1 Hz, 2H), 4.64 (s, 1H), 4.08 (d, *J* = 15.6 Hz, 1H), 4.04 (d, *J* = 15.8 Hz, 1H), 3.98 (t, *J* = 6.5 Hz, 1H), 2.89 (dd, *J* = 16.4, 7.3 Hz, 1H), 2.56 (dd, *J* = 16.4, 5.8 Hz, 1H), 2.27 (s, 3H). LC-MS (ESI, +ve) *m/z*: [M + H]$^+$ calcd for $C_{20}H_{16}N_4O_2S_2$ 408.07; found, 409.1.

Chiral resolution of **484**: mobile phase: EtOH : n-Hexane = 90 : 10; flow rate of 0.5 mL/min; retention time: (*R*)-**484** 11.4 min and (*S*)-**484** 14.9 min.

X-ray crystallography study of (*R*)-**484**

A single crystal of (*R*)-**484** was grown by slow evaporation of solution of 5 mg of (*R*)-**484** in 2 mL of THF at room temperature. The stereochemistry of (*R*)-**484** was determined by D8 Venture TXS PHOTON Ⅱ.

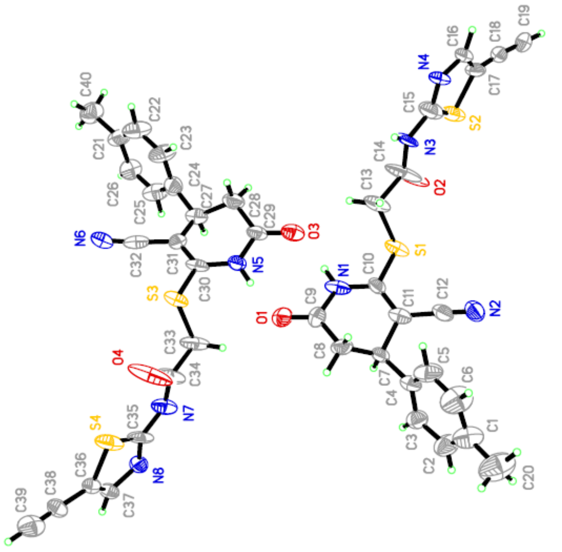

Scheme 3. Synthesis of photo-affinity probe **496**[a]

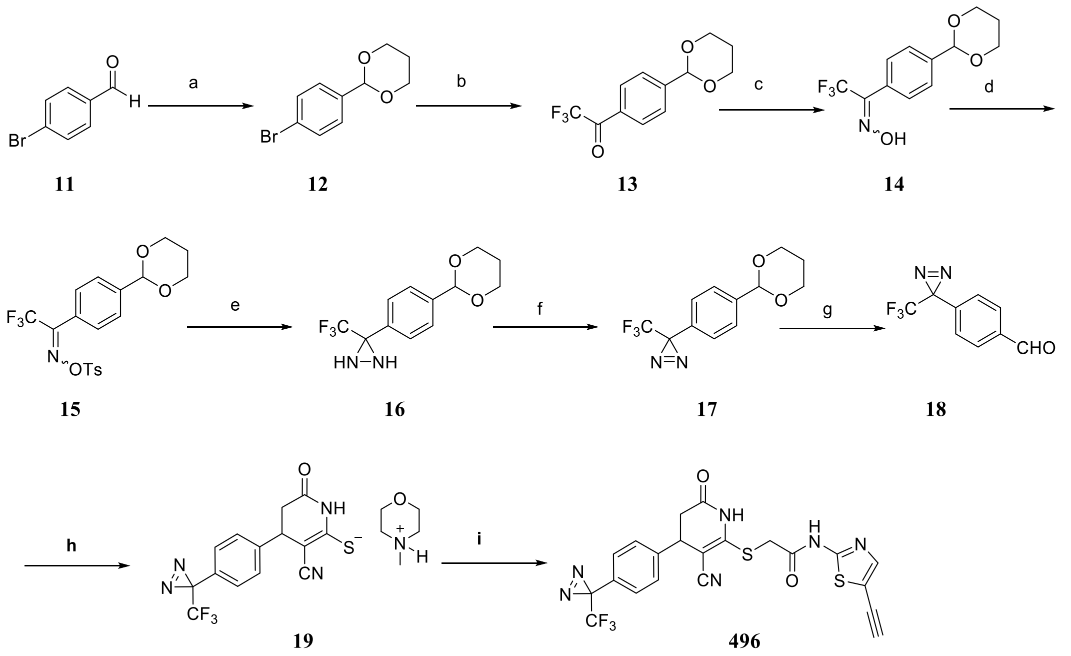

Reagents and conditions: (a) 1,3-propanediol, $MeSO_3H$, toluene, r.t., 4h; (b) n-BuLi, CF_3CO_2Et, THF, -78 °C, 1h; (c) $NH_2OH·HCl$, pyridine/EtOH, 65 °C, 3 h; (d) TsCl, Et_3N, DMAP, DCM, r.t., overnight; (e) liquid NH_3 , -78 °C - r.t., 50 h; (f) I_2, Et_3N, MeOH, rt, 3h; (g) 0.5 M H_2SO_4 (aq), acetone/water, r.t., overnight; (h) 2-cyanothioacetamide, Meldrum's acid, 4-methylmorpholine, EtOH, r.t. - reflux, 6 h; (i) **10**, tetrabutylammonium iodide, DMA, r.t., 10 min.

Step 1 2-(4-bromophenyl)-1,3-dioxane **(12)**.

To a solution of **11** (3.00 g, 16.30 mmol) and 1,3-propanediol (1.36 g, 17.93 mmol) in toluene (30 mL) was added $MeSO_3H$ (0.05 g, 0.49 mmol). After stirring at r.t. for 4 h, the reaction mixture was concentrated. The residue was partitioned between saturated $NaHCO_3$ (aq) (10 mL) and EtOAc (20 mL). The aqueous phase was further extracted with EtOAc (20 mL). The combined EtOAc extracts were dried over anhydrous Na_2SO_4, and concentrated under reduced pressure to afford the title compound **12** (3.80 g, 96.4%) as an oil. ^1H NMR (400

MHz, $CDCl_3$) δ 7.49 (d, *J* = 8.4 Hz, 2H), 7.36 (d, *J* = 8.5 Hz, 2H), 5.46 (s, 1H), 4.26 (dd, *J* = 10.8, 5.0 Hz, 2H), 3.98 (td, *J* = 12.3, 2.3 Hz, 2H), 2.21 (qt, *J* = 12.6, 5.0 Hz, 1H), 1.51-1.39 (m, 1H). LC-MS (ESI, +ve) *m/z*: $[M + H]^+$ calcd for $C_{10}H_{11}BrO_2$ 241.99 and 243.99; found, 243.0 and 245.0 .

Step 2 1-(4-(1,3-dioxan-2-yl)phenyl)-2,2,2-trifluoroethan-1-one (**13**).

To a solution of **12** (3.58 g, 14.66 mmol) in THF (40 mL) cooled to -78 °C under N_2 was added n-BuLi (8.79 mL, 2.5 M in hexane, 21.99 mmol) dropwise over a period of 15 min. After stirring at -78 °C for 30 min, CF_3CO_2Et (3.12 g, 21.99 mmol) was added. After stirring for additional 15 min at -78 °C, the reaction was quenched with saturated aqueous $NaHCO_3$ (10 mL), followed by H_2O (50 mL). The mixture was extracted with EtOAc (2 x 50 mL). The combined EtOAc extracts were dried over anhydrous Na_2SO_4, and concentrated to afford the title compound **13** (3.80 g, 100%) as an oil. ^1H NMR (400 MHz, $CDCl_3$) δ 8.07 (d, *J* = 8.3 Hz, 2H), 7.67 (d, *J* = 8.3 Hz, 2H), 5.56 (s, 1H), 4.32-4.28 (m, 2H), 4.08-3.95 (m, 2H), 2.31-2.16 (m, 1H), 1.51-1.47 (m, 1H). LC-MS (ESI, +ve) *m/z*: $[M + H]^+$ calcd for $C_{12}H_{11}F_3O_3$ 260.07; found, 261.1 .

Step 3 (*Z*/*E*)-1-(4-(1,3-dioxan-2-yl)phenyl)-2,2,2-trifluoroethan-1-one oxime (**14**).

A solution of **13** (4.24 g, 16.3 mmol) and hydroxylamine hydrochloride (2.24 g, 32.61 mmol) in pyridine/EtOH (30 mL/10 mL) was heated at 65 °C for 3 h. The reaction mixture was then concentrated under reduced pressure, and the residue was dissolved in DCM (100 mL), washed with water (50 mL) and brine (50 mL). The DCM phase was dried over anhydrous Na_2SO_4 and concentrated under reduced pressure to afford the crude title compound **14** which

was used for next step without further purification. LC-MS (ESI, +ve) *m/z*: $[M + H]^+$ calcd for $C_{12}H_{12}F_3NO_3$ 275.08; found, 276.1 .

Step 4 (*Z*/*E*)-1-(4-(1,3-dioxan-2-yl)phenyl)-2,2,2-trifluoroethan-1-one O-tosyl oxime (**15**).

To a solution of **14** (5.00 g, 18.18 mmol) in DCM (50 mL) was added Et_3N (6.43 g, 63.63 mmol), DMAP (0.11 g, 0.91 mmol) and TsCl (6.91 g, 36.36 mmol). After stirring at r.t. overnight, the reaction mixture was washed with saturated aqueous $NaHCO_3$ (50 mL) and brine (50 mL). The DCM phase was dried over anhydrous Na_2SO_4, concentrated under reduced pressure. The residue was purified by silica gel column chromatography (PE : EA=3 : 1) to afford the title compound **15** (7.02 g, 90%, 1:1 mixture of *Z*/*E* isomers) as a yellow solid. ^1H NMR (400 MHz, $CDCl_3$) δ 7.89-7.86 (m, 2H), 7.60 (d, *J* = 8.2 Hz, 1H), 7.54 (d, *J* = 8.2 Hz, 1H), 7.47-7.32 (m, 4H), 5.53/5.52 (s, 1H), 4.30-4.25 (m, 2H), 4.03-3.97 (m, 2H), 2.48/2.45 (s, 3H), 2.29-2.15 (m, 1H), 1.51-1.43 (m, 1H). LC-MS (ESI, +ve) *m/z*: $[M + H]^+$ calcd for $C_{19}H_{18}F_3NO_5S$ 429.09; found, 430.9 .

Step 5 3-(4-(1,3-dioxan-2-yl)phenyl)-3-(trifluoromethyl)diaziridine (**16**).

A 100 mL autoclave was charged with **15** (2.00 g, 4.66 mmol) and DCM (5 mL). Approximately 40 mL of liquid NH_3 was condensed into the autoclave at -78 °C. After 2 h at -78 °C, the mixture allowed to stirred at r.t. for two days. NH_3 was then carefully released, and DCM (50 mL) was added to the mixture. The DCM solution was washed with water (2 x 50 mL), dried over anhydrous Na_2SO_4 and concentrated to afford the title compound **16** (1.27 g, 100%) as a pale-yellow oil. ^1H NMR (400 MHz, $CDCl_3$) δ 7.62 (d, *J* = 8.2 Hz, 2H), 7.55 (d, *J* = 8.2 Hz, 2H), 5.52 (s, 1H), 4.35-4.19 (m, 2H), 4.03-3.96 (m, 2H), 2.78 (d, *J* = 8.8 Hz, 1H),

2.28-2.16 (m, 2H), 1.46 (dtt, *J* = 12.3, 2.5, 1.4 Hz, 1H). LC-MS (ESI, +ve) *m/z*: $[M + H]^+$ calcd for $C_{12}H_{13}F_3N_2O_2$ 274.09; found, 275.1.

Step 6 3-(4-(1,3-dioxan-2-yl)phenyl)-3-(trifluoromethyl)-3H-diazirine (**17**).

A solution of **16** (1.20 g, 4.37 mmol), I_2 (1.33 g, 5.26 mmol) and Et_3N (1.33 g, 13.14 mmol) in MeOH (15 mL) was stirred at r.t. for 3 h in dark. EtOAc (50 mL) and water (50 mL) were added to the mixture. A saturated aqueous solution of $Na_2S_2O_3$ was added until the color of the mixture faded. The EtOAc phase was separated, washed with brine (30 mL), dried over anhydrous Na_2SO_4 and concentrated to afford compound **17** (1.14 g, 96%) as a pale-yellow oil. ^{1}H NMR (400 MHz, $CDCl_3$) δ 7.52 (d, *J* = 8.4 Hz, 2H), 7.20 (d, *J* = 8.3 Hz, 2H), 5.50 (s, 1H), 4.26 (dd, *J* = 10.8, 5.0 Hz, 2H), 3.98 (td, *J* = 12.3, 2.3 Hz, 2H), 2.21 (qt, *J* = 12.5, 5 Hz, 1H), 1.51-1.38 (m, 1H). LC-MS (ESI, +ve) m/z: $[M + H]^+$ calcd for $C_{12}H_{11}F_3N_2O_2$ 272.08; found, 273.1 .

Step 7 4-(3-(trifluoromethyl)-3H-diazirin-3-yl)benzaldehyde (**18**).

To a solution of **17** (0.80 g, 2.94 mmol) in acetone (60 mL) cooled at 0 °C was added a solution of H_2SO_4 (29.4 mL, 0.5 M) dropwise. After stirring at r.t. overnight, the pH of the reaction mixture was adjusted to 8 with a saturated solution of $NaHCO_3$. The mixture was then extracted with EtOAc (3 x 50 mL). The combined EtOAc extracts were dried over anhydrous Na_2SO_4 and concentrated to afford the title compound **18** (0.60 g, 95.38%) as a colorless oil. ^{1}H NMR (400 MHz, $CDCl_3$) δ 10.05 (s, 1H), 7.92 (d, *J* = 8.2 Hz, 2H), 7.35 (d, *J* = 8.2 Hz, 2H). LC-MS (ESI, +ve) m/z: $[M + H]^+$ calcd for $C_9H_5F_3N_2O$ 214.04; found, none.

Step 8 (*R*/*S*)-4-methylmorpholin-4-ium3-cyano-6-oxo-4-(4-(3-(trifluoromethyl)-3H-diazirin-3-yl)phenyl)-1,4,5,6-tetrahydropyridine-2-thiolate (**19**).

A solution of **18** (0.20 g, 0.93 mmol) and 2-cyanothioacetamide (0.094 g, 0.93 mmol) and 4-methylmorpholine (0.040 g, 0.39 mmol) in EtOH (10 mL) was stirred at r.t. for 1 h. Meldrum's acid (0.014 g, 0.97 mmol) and 4-methylmorpholine (0.10 g, 0.99 mmol) were then added to the mixture. After stirring at r.t. for 1 h, the mixture was heated to reflux for 4 h under N_2. The reaction mixture was cooled to r.t. and concentrated under reduced pressure to afford crude compound **19** which was used for next step without further purification. LC-MS (ESI, -ve) *m/z*: $[M - H]^-$ calcd for $C14H8F3N4OS^-$ 337.04 ; found, 337.0 .

Step 9 (*R*/*S*)-2-((3-cyano-6-oxo-4-(4-(3-(trifluoromethyl)-3H-diazirin-3-yl)phenyl)-1,4,5,6-tetrahydropyridin-2-yl)thio)-*N*-(5-ethynylthiazol-2-yl)acetamide (**496**).

A solution of **19** (0.021 g, 0.47 mmol), **10** (0.094 g, 0.47 mmol) and tetrabutylammonium iodide (0.017 g, 0.05 mmol) in DMA (1 mL) was stirred at r.t. for 10 min. Water (10 mL) was added to the reaction mixture and the mixture was extracted with EtOAc (2 x 10 mL). The combined EtOAc extracts were washed with brine, dried over anhydrous Na_2SO_4 and concentrated. The residue was purified by silica gel column chromatography (PE : EtOAc = 1 : 1) and was further purified by Prep-HPLC (H_2O (containing 0.1% formic acid) : MeCN = 50 : 50) to afford **496** (0.020 g, 8.5%) as a pale-yellow solid. ^1H NMR (400 MHz, DMSO-*d₆*) δ 12.80 (s, 1H), 10.73 (s, 1H), 7.79 (s, 1H), 7.38 (d, *J* = 8.4 Hz, 2H), 7.26 (d, *J* = 8.2 Hz, 2H), 4.64 (s, 1H), 4.12-4.10 (m, 1H), 4.08 (d, *J* = 15.2 Hz, 1H), 4.04 (d, *J* = 15.2 Hz, 1H), 2.94 (dd, *J* = 16.4, 7.3 Hz, 1H), 2.59 (dd, *J* = 16.4, 6.0 Hz, 1H). ^{19}F NMR (376 MHz, DMSO-d_6) δ -64.55 (s). LC-MS (ESI, +ve) *m/z*: $[M + H]^+$ calcd for $C_{21}H_{13}F_3N_6O_2S_2$ 502.05 ; found, 502.9 .

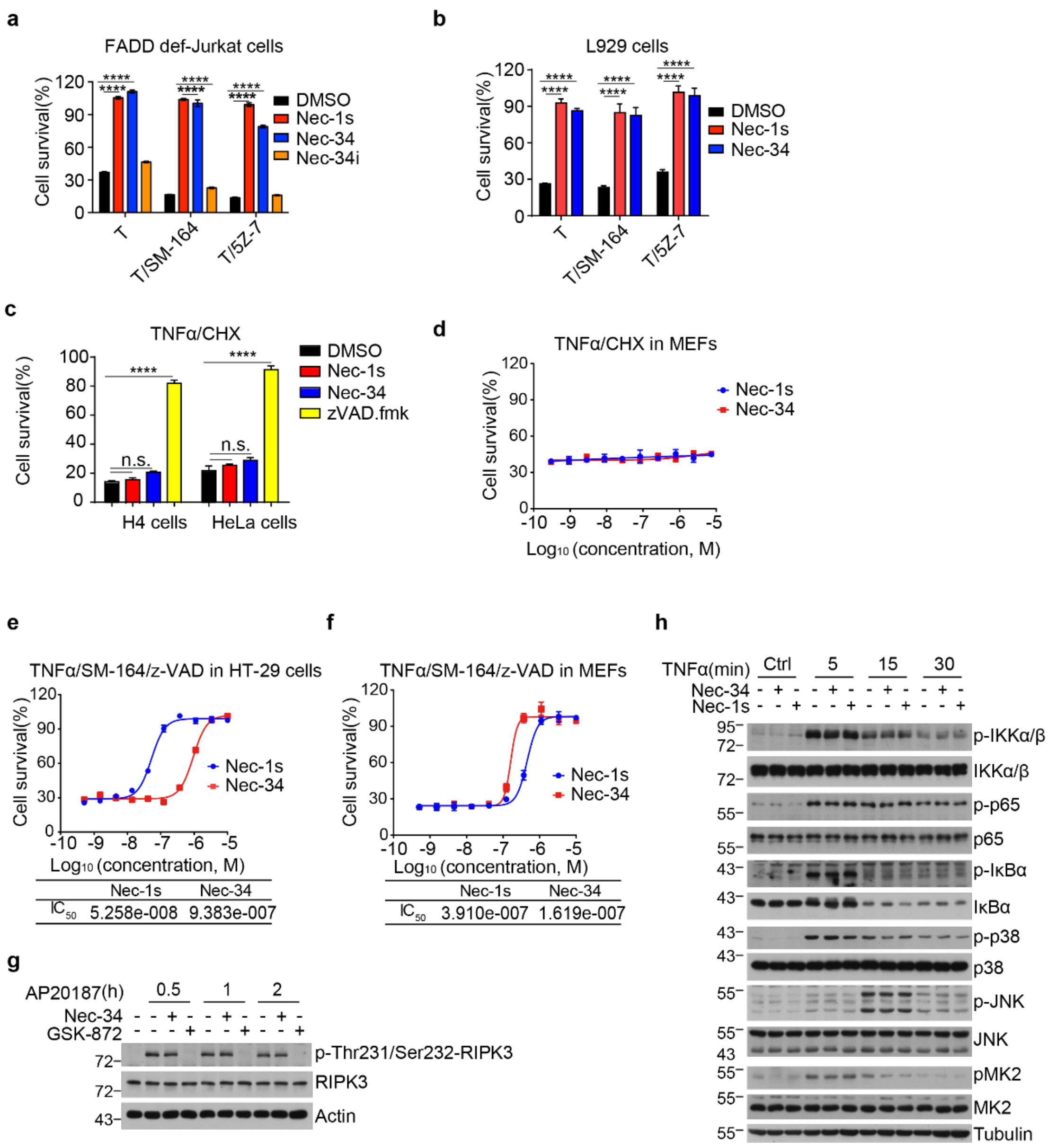

Supplementary Fig. S1 Nec-34 inhibits necroptosis of human and mouse cells.

a,**b** FADD deficient Jurkat cells (**a**) and L929 cells (**b**) were pretreated with 10 µM Nec-1s, Nec-34 or Nec-34i for 30 min and then treated with 25 nM SM-164 or 100 nM 5Z-7 for 2 h as indicated, 20 ng/mL TNFα (T) was then added. The TNFα only group were incubated for 16 h, and the TNFα/SM164 and TNFα/5Z-7 groups were incubated for 8 h. **c** H4 cells and HeLa cells were pretreated with 10µM Nec-1s, Nec-34 or zVAD.fmk for 30 min and then treated with 20 ng/mL TNFα and 1µg/mL CHX for 24h to induce apoptosis as indicated. **d** MEFs were

pretreated with different concentrations of Nec-1s or Nec-34 for 30 min and then treated with 20 ng/mL TNFα and 1μg/mL CHX for 24h to induce apoptosis as indicated. **e**,**f** Dose-response curves of Nec-1s and Nec-34 in cytoprotection of HT-29 cells (**e**) and MEFs (**f**). The cells were pretreated with 10μM Nec-1s or Nec-34 for 30 min and then treated with TNFα/SM164/zVAD.fmk for 12h. **g** RIPK3-FKBP NIH/3T3 cells were pretreated with 10 μM Nec-34 or GSK872 for 30 min and then 2 nM AP20187 was added for additional periods of time. The cell lysates were analyzed by western blotting with indicated antibodies. **h** FADD def Jurkat cells were pretreated with 10 μM Nec-1s or Nec-34 for 30 min and then treated with 100 ng/ml TNFα for various time points. The cell lysates were analyzed by phosphorylated and total IKKα/β, p65, IκBα, p38, JNK, MK2 and actin as indicated. The cell death in **a**-**f** were measured by CellTiter-Glo assays, and the results shown depict mean (±s.e.m.) of n=3 independent biological experiments. P values were calculated by two-tailed Student's t-test (*P<0.05, **P<0.01, ***P<0.001, ****P<0.0001).

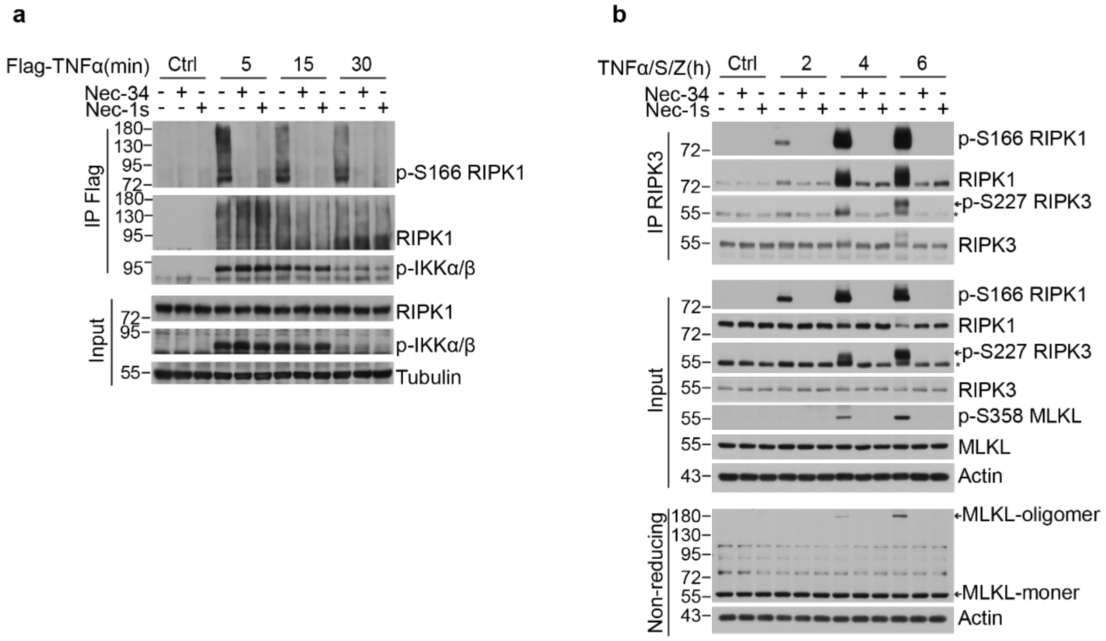

Supplementary Fig. S2 Nec-34 inhibits the formation of complex II.

a FADD def Jurkat cells were pretreated with 10 μM Nec-1s or Nec-34 for 30 min and then treated with 100 ng/mL Flag-TNFα for indicated time points. The cells were lysed with 0.5% Nonidet P-40 buffer and cell lysates were immunoprecipitated with anti-Flag M2 affinity agarose gel. All immunoprecipitated complexes and whole-cell lysates were analyzed by western blotting with indicated antibodies. **b** HT-29 cells were pretreated with 10 μM Nec-1s or Nec-34 for 30 min and then pretreated with 100 nM SM-164 (S) for 2 h and 50 μM zVAD.fmk (Z) for 30 min, and then 50 ng/mL TNFα was added for indicated time points. The cells were lysed with 0.5% Nonidet P-40 buffer and cell lysates were immunoprecipitated with anti-RIPK3 antibody. All immunoprecipitated complexes and whole-cell lysates were analyzed by western blotting with indicated antibodies.

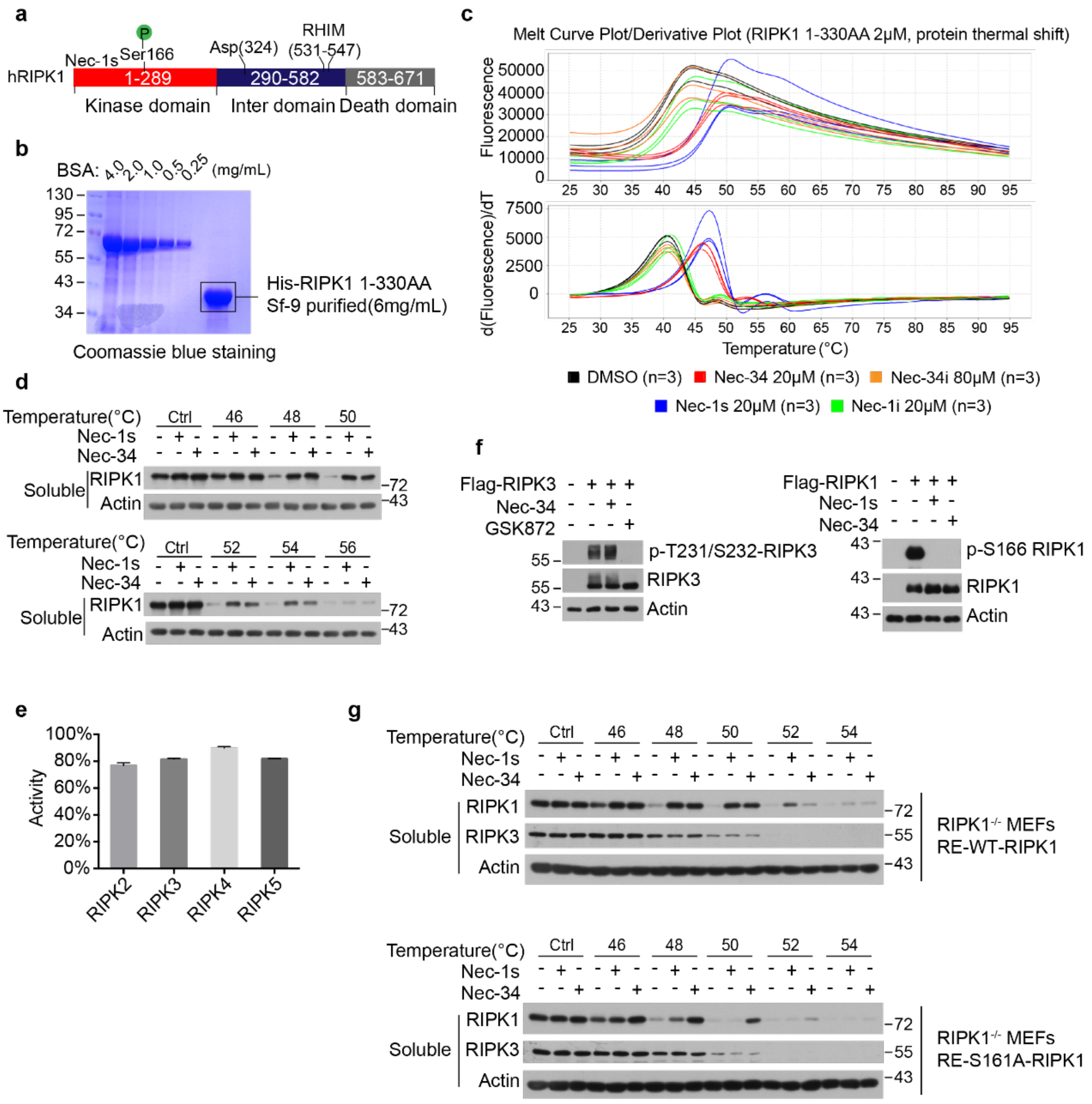

Supplementary Fig. S3 Nec-34 directly binds to RIPK1 and inhibits kinase activity of RIPK1.

a A schematic diagram of human RIPK1. **b** His-RIPK1 (residues 1-330) was purified from Sf-9 cells, and then diluted it to a final concentration of 6mg/mL. 0.25, 0.5, 1.0, 2.0, 4.0 mg/mL BSA were prepared and analyzed by SDS-PAGE and Coomassied blue staining. **c** Thermal stability profiles in protein thermal shift assay. Recombinant kinase domain of hRIPK1 (residues 1-330, 2 μM) purified from Sf-9 cells was treated with 20 μM Nec-34, Nec-1s, Nec-1i or 80 μM Nec-34i for 30min. The protein thermal stability was analyzed using differential

scanning calorimetry by real-time PCR and the melting temperatures were calculated by Protein Thermal Shift™ Software. Three replicates for each reaction were performed. **d** Cellular thermal shift assay. MEFs were treated with 10 μM Nec-34 or Nec-1s for 2 h and then harvested and resuspended with PBS. The cells were incubated at 37, 46, 48, 50, 52, 54, 56°C for 3min, and then frozen in liquid nitrogen quickly. The cells were subject to repeated freeze-thaw three times, and then centrifuged at 20,000g at 4°C for 10min. The soluble part was lysed with 2%SDS buffer and analyzed by western blotting analysis of RIPK1 and actin antibodies as indicated. **e** Nec-34 was measured at a concentration of 10 μM against RIP kinase family members using a radiometric HotSpotSM enzymatic assay by Reaction Biology Corporation. **f** Flag-tagged RIPK3 was overexpressed in 293T cells for 24h, cells were then treatment with 10 μM Nec-34 or GSK872 for 12 h after transfection and lysed with Nonidet P-40 buffer 24 h after transfection (Left). Flag-tagged RIPK1 (residues 1-330) was overexpressed in 293T cells for 24h, and cells were then treated with 10 μM Nec-1s or Nec-34 for 12h after transfection and lysed with Nonidet P-40 buffer 24 h after transfection (Right). The lysates were analyzed by western blotting with indicated antibodies. **g** Cellular thermal shift assay. WT-RIPK1 or S161A-RIPK1 reconstituted $RIPK1^{-/-}$ MEFs were treated with 10 μM Nec-34 or Nec-1s for 2 h and then harvested and resuspended with PBS. The cells were incubated at 37, 46, 48, 50, 52, 54°C for 3min, and then frozen in liquid nitrogen quickly. The cells were subject to repeated freeze-thaw three times, and then centrifuged at 20,000g at 4°C for 10min. The soluble part was lysed with 2%SDS buffer and analyzed by western blotting analysis of RIPK1 and actin antibodies as indicated.

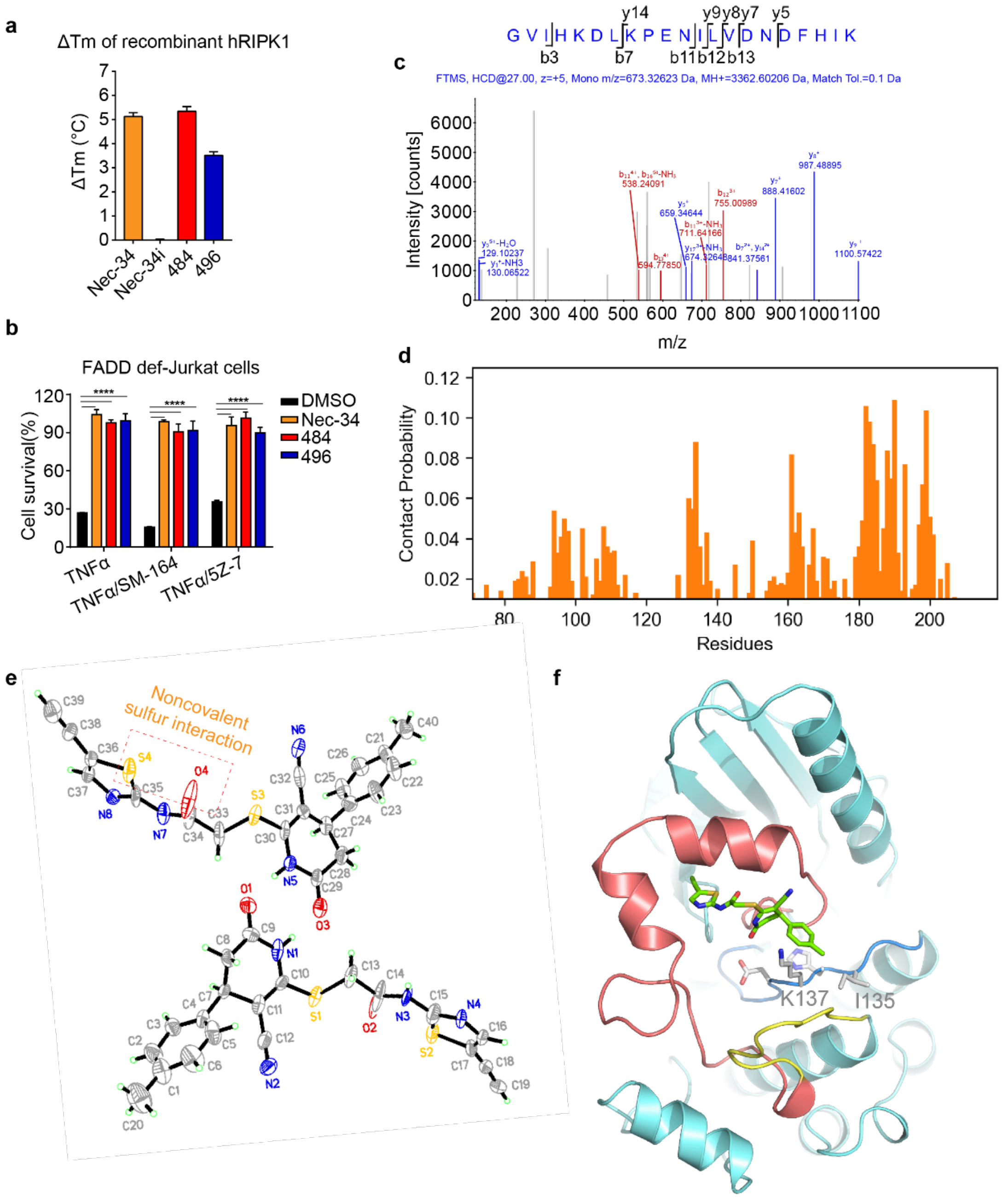

Supplementary Fig. S4 Identification of the Nec-34 binding motifs in RIPK1 kinase.

a The melting temperature (Tm) of recombinant hRIPK1 in Nec-34, Nec-34i, **496** or **484** treatment groups were compared to control group and presented as ΔTm for protein thermal shift assay. Recombinant hRIPK1 (residues 1-330, 2 μM) purified from Sf-9 cells was treated with 80 μM Nec-34, Nec-34i, **484** or **496** for 30min. The protein thermal stability was analyzed

through the differential scanning calorimetry by real-time PCR and the melting temperatures were calculated by Protein Thermal Shift™ Software. Three replicates for each reaction were performed. **b** Inhibition of necroptosis by Nec-34, **484** and **496**. FADD deficient Jurkat cells were pretreated with 10 μM Nec-34, **484** or **496** for 30 min and then treated with 25 nM SM-164 or 100 nM 5Z-7 for 2 h as indicated, cells were further treated with 20 ng/mL TNFα. The TNFα only group were incubated for 16 h, the TNFα/SM164 and TNFα/5Z-7 groups were incubated for 8 h. **c** MS/MS of a hRIPK1 peptide 133-GVIHKDLKPENILVDNDFHIK-153 crosslinked with biotinylated compound **496** as shown in Fig 4**c**. The ion b3, b7, b11, b12, b13 are annotated with a mass shift +918.2587 Da. **d** The protein−ligand contact probability for RIPK1 residues obtained from GaMD enhanced sampling simulations of RIPK1 and compound **484**. A contact is defined if the shortest heavy atom distance between a residue and ligand is smaller than 4.5 Å, and residues with higher contact probability show higher possibility to constitute the ligand binding site. **e** Single crystal of structure of compound **484**. **f** Cartoon mode of predicted bingding mode of **484** in Fig 4**d**. Lys137 and Ile135 are highlighted in gray sticks. The cell death in **b** was measured by CellTiter-Glo assays, results shown depict the mean (±s.e.m.) of n=3 independent biological experiments. P values were calculated by two-tailed Student's t-test (*P<0.05, **P<0.01, ***P<0.001, ****P<0.0001).

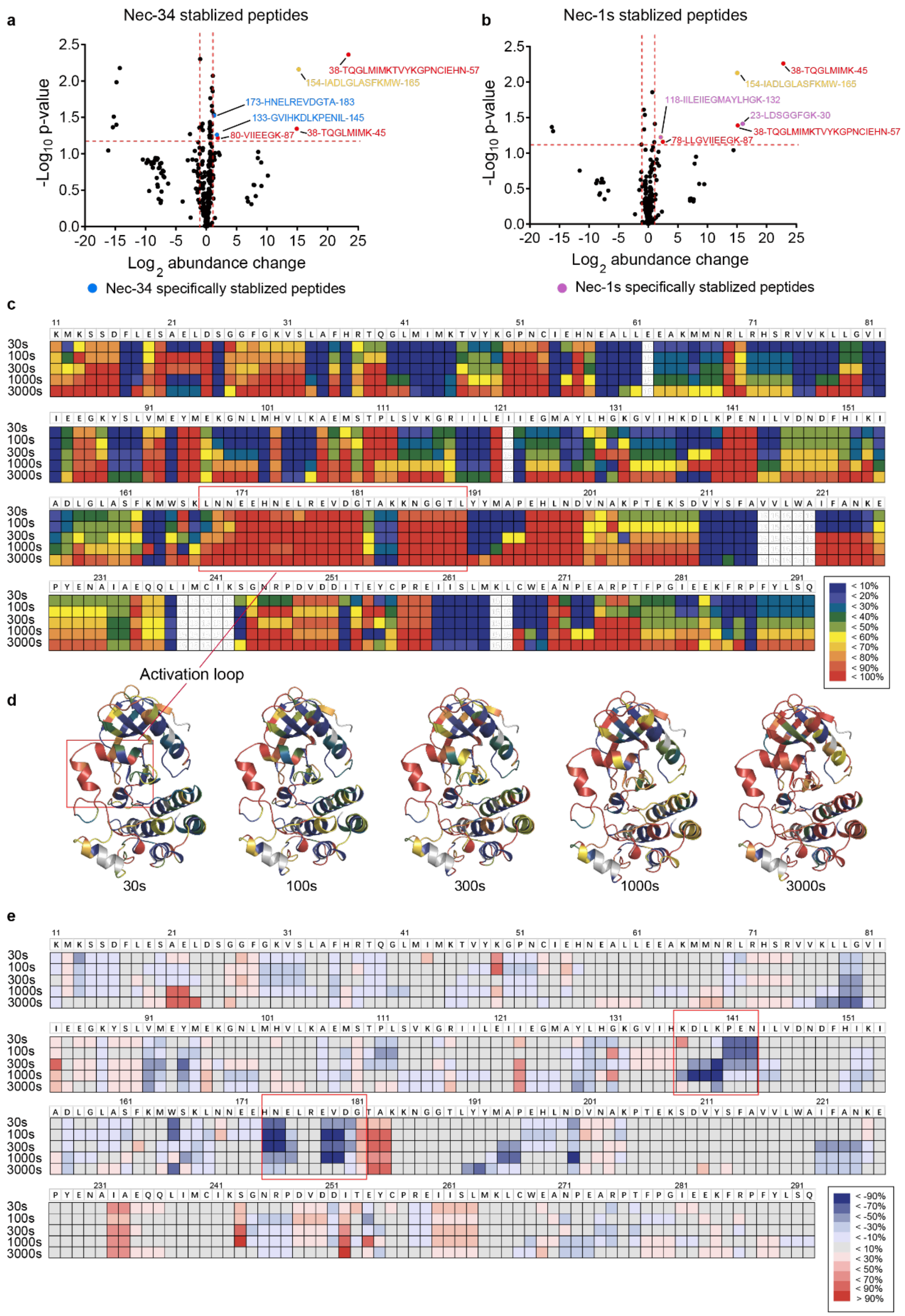

Supplementary Fig. S5 Validation of the Nec-34 binding sites in RIPK1 kinase .

a-b Volcano plot showing peptides identified by LiP-MS. Recombinant hRIPK1 (residues 1-

330, 20 μg) purified from Sf-9 cells was treated with DMSO, Nec-1s (200μM) and Nec-34 (200μM) respectively for 2 h at room temperature and then subjected to LiP-MS analysis (details was shown in Materials and Methods section). Abundance change of peptides in Nec-34 treatment group (**a**) and Nec-1s treatment group (**b**) compared to control group was presented as volcano plots. The x-axis shows Log_2 fold changes and the y-axis shows $-log_{10}P$ values. Stabilized LiP-peptides were cherry picked in Nec-1s or Nec-34 treatment group with p-value<0.05 and abundance change>2 and further categorized into different subgroups. Nec-34 specifically stabilized peptides were colored blue, Nec-1s specifically stabilized peptides were colored magenta. Peptide both stabilized by Nec-34 and Nec-1s in the adjacent region was colored yellow. Peptides both stabilized by Nec-34 and Nec-1s in the distal region was colored red. **c** HDX-MS analysis to determine the deuterium uptake spectrum of free RIPK1. Recombinant hRIPK1(residues 1-330, 100μg) purified from Sf-9 cells was treated with DMSO or Nec-34 (200μM) respectively for 2 h at room temperature and then subjected to HDX-MS analysis (details was shown in Materials and Method section). The deuterium uptake spectrums of free RIPK1 at 30s, 100s, 300s, 1000s and 3000s were indicated by a color gradient from blue (<10% deuterium uptake) to red (>90% deuterium uptake). **d**. The deuterium uptake spectrums of free RIPK1 in **c** were mapped to the predicted bindging mode of **484** and indicated by a color gradient from blue (<10% deuterium uptake) to red (>90% deuterium uptake). **e** HDX-MS analysis of Nec-34 binding sites. Summarized data for deuterium uptake differences between Nec-34 bound RIPK1 and free RIPK1 at 30s, 100s, 300s, 1000s and 3000s were indicated by a color gradient from blue (decreased deuterium uptake) via white (unchanged) to red (increased deuterium uptake).

a

	TNFα treatment			TSZ treatment		
		Nec-1s	Nec-34		Nec-1s	Nec-34
Human cells	FADD def Jurkat cells IC_{50} (M)	7.389e-008	6.672e-007	HT-29 cells IC_{50} (M)	5.258e-008	9.383e-007
Murine cells	L929 cells IC_{50} (M)	2.467e-007	1.346e-007	MEFs IC_{50} (M)	3.910e-007	1.619e-007

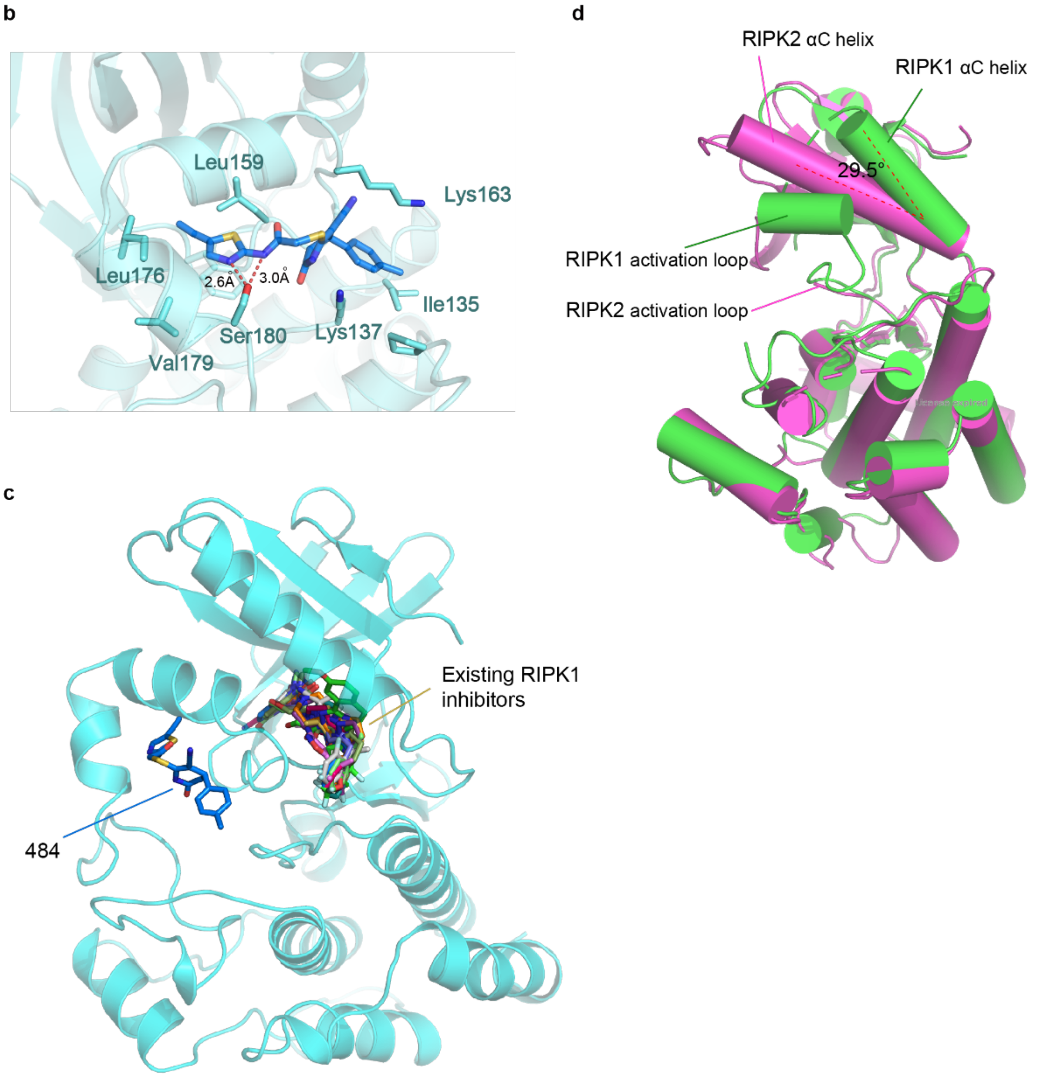

Supplementary Fig. S6 Mutagenesis studies of Nec-34 binding sites in RIPK1 kinase.

a A summarized IC_{50} table of Nec-1s and Nec-34 in human and murine cells with indicated treatments from Fig. 1b-c and Supplementary Fig. S1e-f. **b** A closed-up view of the **484** binding sites of RIPK1 according to predicted binding mode of **484** in Figure. 4d. The residue Asp180 was mutated to Ser by employing PyMOL tool. **484** was shown in blue sticks, the binding sites of **484** in D180S mutant were shown in cyan sticks. The H-bond was shown in red dashed line.

c Alignment of computational binding mode of compound **484** in Fig. 4d with the co-crystal structure of RIPK1 and all of its reported inhibitors (PDB: 4ITH, 4ITI, 4ITJ, 5HX6, 6HHO, 6NYH, 6C3E, 6C4D, 6OCQ, 6R5F, 6RLN, 5TX5, 4NEU, 6NW2). RIPK1 was colored cyan, compound **484** was shown in blue sticks, the reported RIPK1 inhibitors were shown in sticks with different colors. **d** Protein structure comparison of RIPK1 (PDB: 4ITH) and RIPK2 (PDB: 4C8B). RIPK1 was colored green, RIPK2 was colored magenta. The rotation angle of αC helix between RIPK1 and RIPK2 was shown in red dashed line.

a

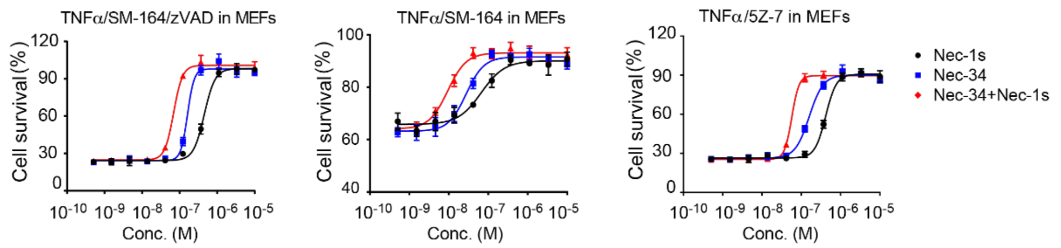

b

CI (combination index) values at ED 90			
	TNFα/SM164/zVAD	TNFα/SM164	TNFα/5Z7
MEFs	0.11525	0.23906	0.30997

CI<1 synergism; CI=1 additive effect; CI>1 antagonism

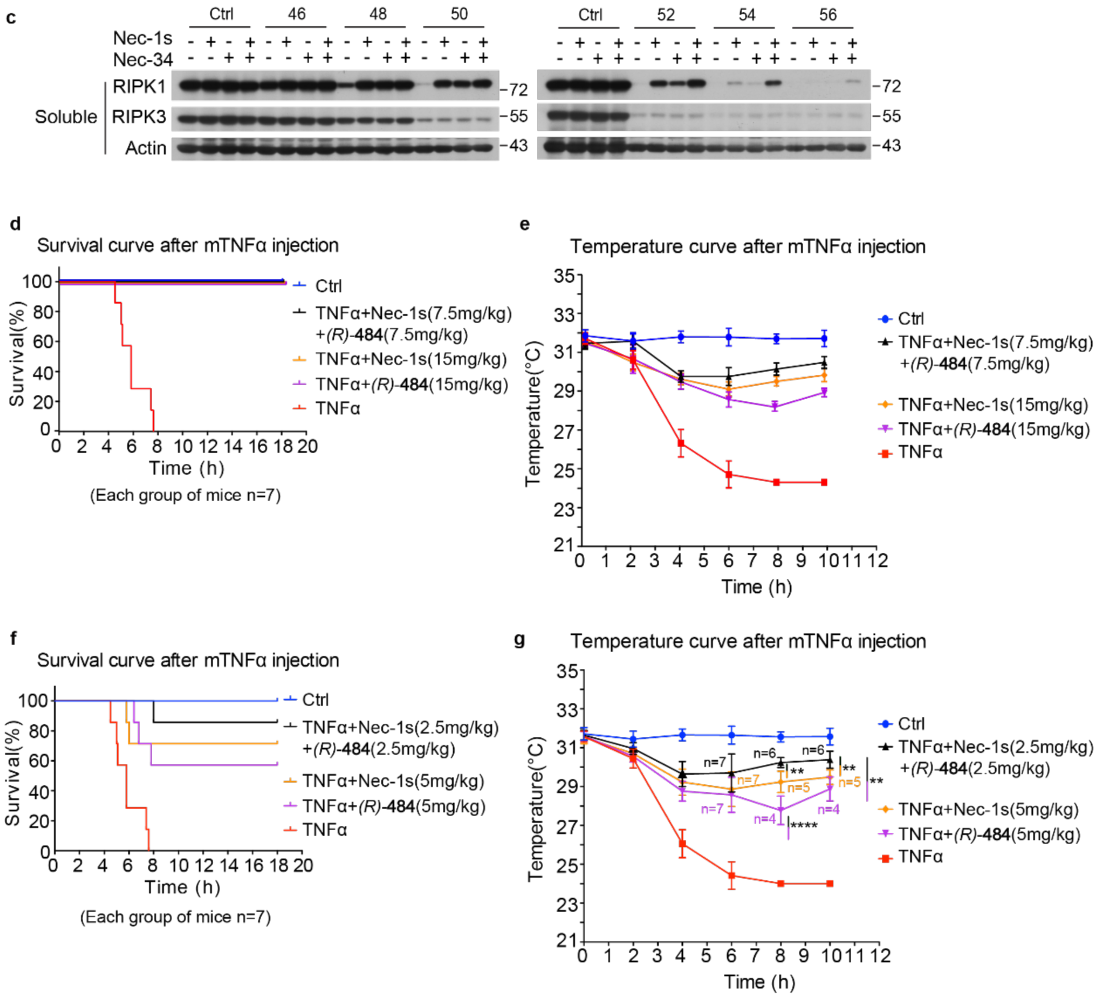

Supplementary Fig. S7 Synergistic effects of Nec-1s and Nec-34 in cells and *in vivo*.

a Synergistic inhibitory effects of Nec-1s and Nec-34 in MEFs. Cells were pretreated with different concentrations of Nec-1s, Nec-34, or Nec-1s/Nec-34 at a constant ratio 1:1 (e.g. 10

μM Nec-1s alone, 10 μM Nec-34 alone or 5 μM+5 μM Nec-1s/Nec-34) for 30 min and then TNFα/SM-164/zVAD.fmk, TNFα/SM-164 or TNFα/5Z-7 were added for an additional 8 h. **b** The combination indexes calculated from (**a**). **c** Synergistic effects of Nec-34 and Nec-1s in cellular thermal shift assay. MEFs were treated with 10 μM Nec-34, 10 μM Nec-1s or combination of Nec-34 and Nec-1s (5 μM + 5 μM) for 2 h and then harvested and resuspended with PBS. The cells were incubated at 37, 46, 48, 50, 52, 54, 56°C for 3min, and then frozen in liquid nitrogen quickly. The cells were subject to repeated freeze-thaw three times, and then centrifuged at 20,000g at 4°C for 10min. The soluble part was lysed with 2%SDS buffer and analyzed by western blotting analysis of RIPK1 and actin antibodies as indicated. **d**,**e** TNFα-induced SIRS model. Six-week-old C57BL/6J male mice were pretreated intragastrically with Nec-1s (15 mg/kg, n=7), **484** (15 mg/kg, n=7) or combination of Nec-1s and **484** (7.5 mg/kg+7.5 mg/kg, n=7) for 15min, and then intravenously injected with mTNFα (0.5μg per g mouse body weight, diluted with endotoxin-free PBS). Control mice were injected with vehicle only (endotoxin-free PBS, n=7). Survival periods were recorded within 18 h after injection (**d**). Surface body temperature was recorded within 10 h period by an infrared thermometer (**e**) and the body temperature of dead mice was set to room temperature (24℃). **f**,**g** TNFα-induced SIRS model. Six-week-old C57BL/6J male mice were pretreated intragastrically with Nec-1s (5 mg/kg, n=7), **484** (5 mg/kg, n=7) or combination of Nec-1s and **484** (2.5 mg/kg+2.5 mg/kg, n=7) for 15min, and then intravenously injected with mTNFα (0.5μg per g mouse body weight, diluted with endotoxin-free PBS, n=7). Control mice were injected with vehicle only (endotoxin-free PBS). Survival periods were recorded within 18 h after injection (**f**). Surface body temperature was recorded within 10 h period by an infrared thermometer (**g**) and the body

temperature of dead mice was set to room temperature (24℃). P values of body temperatures were calculated by two-tailed Student's t-test $^{**}p < 0.01$,$^{***}p < 0.001$, $^{****}p < 0.0001$. The cell death in **a** was measured by CellTiter-Glo assays. The results shown depict mean (±s.e.m.) of n=3 independent biological experiments. P values were calculated by two-tailed Student's t-test (*P<0.05, **P<0.01, ***P<0.001, ****P<0.0001).

Cpd	FADD-def Jurkat cells			L929		
	TNFα IC_{50}(μM)	TNFα+SM164 IC_{50}(μM)	TNFα+5Z7 IC_{50}(μM)	TNFα IC_{50}(μM)	TNFα+SM164 IC_{50}(μM)	TNFα+5Z7 IC_{50}(μM)
Nec-34	0.622	2.874	1.295	0.084	0.221	0.204
484	0.291	2.200	1.119	0.299	0.316	0.193
(*R*)-484	0.169	1.564	0.519	0.125	0.173	0.077
(*S*)-484	4.672	5.979	21.350	1.820	2.000	2.284
Nec-1s	0.078	0.188	0.288	0.173	0.228	0.279

Supplementary Table. S1 Structure-activity relationship (SAR) of Nec-34.

The IC_{50}s of Nec-1s, Nec-34, and **484** , (*R*)-**484**, (*S*)-**484** in necroptosis FADD def-Jurkat cells and L929 cells were measured by 10-points 2-fold dilution series concentration response assay. Cells were pretreated with different concentrations of Nec-1s, Nec-34, **484**, (*R*)-**484**, (*S*)-**484** for 30 min and then with TNFα for 12 h, or TNFα/SM-164 for 8 h, or TNFα/5Z-7 for 8 h respectively. The cell survival was measured by CellTiterGlo. Results shown are averages of triplicates ± SEM. (*R*) or (*S*) represents a chiralilty of **484**.

	Animal No.	$T_{1/2}$ (h)	T_{max}(h)	C_{max}(ng/mL)
30mg/kg	1	4.22	0.25	286
	2	4.29	1.00	532
	3	5.69	0.25	674
	Mean	4.73	0.50	497
	SD	0.83	0.43	196
100mg/kg	1	4.34	0.25	8008
	2	4.04	1.00	10818
	3	20.6	1.00	2921
	Mean	9.66	0.75	7249
	SD	9.48	0.433	4003

Supplementary Table. S2 Pharmacokinetics parameters of 484 in male CD-1 mouse (n = 3).

A 1:3 mixture (w/w) of **484** with copovidone was prepared by evaporating a solution of the mixture in EtOH (0.1 g of **484** and 0.3 g of copovidone in 30 mL of EtOH). The resulted solid was dissolved in 0.5% HPMC and was given to CD-1 mice (male, 18 – 22 g, n = 3) by p.o. administration at a dosage of 30 mg/kg and 100 mg/kg. Plasma samples were collected at 0.25, 0.5, 1, 2, 4, 8, and 24 h after administration (anticoagulant: EDTA-Na_2). 100 μL of solvent of acetonitrile/methonal（1/1，v/v） with internal standard was added to 10 μL of plasma and vortexed thoroughly. After the mixture was centrifuged for 5 min at 11000 rpm, 35 μL of the supernatant was mixed with 35 μL of water for analysis. Samples were analyzed by 6500 triple quadrupole mass spectrometer (Sciex, USA). An Acquity UPLC BEH C18 (1.7 μm, 50 mm × 2.1 mm, Waters, USA) column was used for the analysis. Gradient elutions were used with a mobile phase composed of solvent A (water containing 0.1% formic acid and 5mM NH_4OAc) and solvent B (acetonitrile containing 0.1% formic acid). The value of AUC_{last}, $AUC_{INF\ obs}$ and $MRT_{INF\ obs}$ were calculated from time - concentration curves in each animal using Phoenix

WinNonlin (CERTARA, USA). C_{max} was determined as the maximum plasma concentration, and T_{max} was the time to reach the maximum concentration.

Time (h)	Plasma Conc. (ng/mL), mean	Brain Conc. (ng/g), mean	Plasma/Brain Ratio*
1	855	48.9	0.0542
4	266	15.7	0.0642
12	5.51	0.667	0.402

* Average ratio of 3 mice.

Supplementary Table. S3 The concentration of 484 in plasma and brain after p.o. administration.

Compound **484** (30 mg/kg, 10 mL/kg) was dissolved in PEG400 and H_2O (1/1, v/v), and was given to CD-1 mice (male, 18-22 g, n = 3 for each time point) by p.o. administration. Mice were sacrificed by carbon dioxide at 1 h, 4 h and 12 h post dosing. Plasma and brain samples were collected and the compound concentration in plasma was measured as described above, and the brain samples were prepared via homogenization with 5 times volume of acetonitrile/methanol (1 : 1, v/v). Samples were analyzed by 6500 triple quadrupole Mass Spectrometer (Sciex, USA). An Acquity UPLC BEH C18 column (1.7 µm, 50 mm × 2.1 mm, Waters, USA) was used for the analysis. Gradient elution was performed with a mobile phase composed of solvent A (water containing 0.1% formic acid and 5 mM NH_4OAc) and solvent B (acetonitrile containing 0.1% formic acid).
